# Mixed-Culture Fermentation of Coffee Pulp Induces Metabolomic Changes and Enhances Multi-Target Bioactivity Relevant to Androgenetic Alopecia

**DOI:** 10.3390/ijms27177865

**Published:** 2026-09-02

**Authors:** Anurak Muangsanguan, Warintorn Ruksiriwanich, Niphawan Panti, Kasirawat Sawangrat, Pattarapa Pummara, Pornchai Rachtanapun, Korawan Sringarm, Sarana Rose Sommano, Sucheewin Krobthong, Chaiwat Arjin, Apinya Satsook, Juan Manuel Castagnini

**Affiliations:** 1 Department of Pharmaceutical Sciences, Faculty of Pharmacy, Chiang Mai University, Chiang Mai 50200, Thailand; anurak_m@cmu.ac.th (A.M.); kasirawat.s@cmu.ac.th (K.S.); 2 Cluster of Valorization and Bio-Green Transformation for Translation Research Innovation of Raw Materials and Products, Chiang Mai University, Chiang Mai 50200, Thailand; korawan.s@cmu.ac.th (K.S.); sarana.s@cmu.ac.th (S.R.S.); 3 Cluster of Agro Bio-Circular-Green Industry (Agro BCG), Chiang Mai University, Chiang Mai 50200, Thailand; niphawan.p@cmu.ac.th (N.P.); pornchai.r@cmu.ac.th (P.R.); 4 Faculty of Agro-Industry, Chiang Mai University, Chiang Mai 50100, Thailand; pattarapa_pummara@cmu.ac.th; 5 Department of Animal and Aquatic Sciences, Faculty of Agriculture, Chiang Mai University, Chiang Mai 50200, Thailand; chaiwat.arjin@cmu.ac.th; 6 Department of Plant and Soil Sciences, Faculty of Agriculture, Chiang Mai University, Chiang Mai 50200, Thailand; 7 Center of Excellence in Natural Products Chemistry (CENP), Department of Chemistry, Faculty of Science, Chulalongkorn University, Bangkok 10330, Thailand; sucheewin.k@chula.ac.th; 8 Office of Research Administration, Chiang Mai University, Chiang Mai 50200, Thailand; apinya.satsook@cmu.ac.th; 9 Research Group in Innovative Technologies for Sustainable Food (ALISOST), Department of Preventive Medicine and Public Health, Food Science, Toxicology and Forensic Medicine, Faculty of Pharmacy, Universitat de València, Avenida Vicent Andrés Estellés s/n, 46100 Burjassot, Spain; juan.castagnini@uv.es

**Keywords:** 5α-reductase, androgenetic alopecia, co-culture fermentation, coffee pulp, hair growth signaling

## Abstract

Androgenetic alopecia (AGA), the most common form of hair loss, is driven by dihydrotestosterone-mediated follicular miniaturization, oxidative stress, and perifollicular inflammation, requiring multi-target intervention. Coffee pulp is an abundant coffee-processing by-product and a potential source of value-added bioactives relevant to AGA. This study investigated whether fermentation with *Saccharomyces cerevisiae*, *Lactobacillus plantarum*, or their mixed culture could remodel coffee pulp composition and enhance biological activities relevant to AGA. Untargeted metabolomics revealed treatment-dependent metabolic remodeling. LP-CP showed the largest number of significantly altered metabolite features (1020; 666 increased and 354 decreased), whereas MIX-CP exhibited a predominantly upward pattern, with 238 of 283 significantly altered features (84.10%) showing increased abundance relative to the unfermented control. Targeted polyphenol analysis showed MIX-CP contained the highest levels of rosmarinic acid and quercetin. In human hair follicle dermal papilla cells, MIX-CP stimulated proliferation, increased fibroblast proliferation in a conditioned-medium model, partially rescued cell viability under potassium-channel inhibition, and attenuated intracellular reactive oxygen species and lipid peroxidation more than monoculture-fermented extracts. At the transcriptional level, MIX-CP downregulated androgen metabolism genes *SRD5A1* and *SRD5A2* alongside pro-regression mediator TGFB1 while upregulating Wnt/β-catenin (*CTNNB1*), Sonic Hedgehog (*SHH*, *SMO*, *GLI1*), and angiogenic (*VEGF*) genes, showing transcript-level modulation comparable to standard hair-loss drugs for most targets. These findings position MIX-CP as a promising multi-target cosmeceutical for AGA management, warranting further in vivo validation.

## 1. Introduction

In recent years, increasing consumer demand for natural and sustainable products has driven growing interest in plant-derived bioactive compounds for cosmetic and therapeutic applications, particularly in the management of hair loss [1]. Androgenetic alopecia (AGA), the most common form of hair loss, is primarily associated with dihydrotestosterone (DHT)-mediated follicular miniaturization, accompanied by inflammation and oxidative stress, which collectively disrupt hair follicle homeostasis and accelerate the transition from the anagen to telogen phase [2]. Effective interventions therefore require multi-target modulation, including inhibition of 5α-reductase (*SRD5A*), suppression of inflammatory mediators, reduction in oxidative stress, and activation of hair growth–related signaling pathways such as Wnt/β-catenin (*CTNNB1*), Sonic Hedgehog (*SHH*, *SMO*, and *GLI1*), and angiogenesis [2].

At the cellular level, DHT binding to androgen receptors in human hair follicle dermal papilla cells (HFDPCs) triggers downstream inflammatory signaling and upregulates transforming growth factor-beta 1 (TGF-β1), which drives apoptotic cascades in follicular epithelial cells and consolidates premature anagen-to-catagen transition, while simultaneously promoting excessive reactive oxygen species (ROS) generation that overwhelms endogenous antioxidant defenses. These converging mechanisms suppress key regenerative pathways, including Wnt/β-catenin signaling—which acts as an upstream activator of the Sonic Hedgehog cascade to coordinate follicular morphogenesis and sustain anagen progression—and vascular endothelial growth factor (VEGF)-mediated angiogenesis, collectively impairing follicular proliferation and accelerating premature entry into the catagen and telogen phases. Current pharmacological treatments, primarily topical minoxidil (a potassium channel opener that promotes follicular vasodilation and prolongs the anagen phase) and oral finasteride (a type II 5α-reductase inhibitor that suppresses dihydrotestosterone synthesis), address only a subset of these mechanisms and are associated with adverse effects, including scalp irritation, sexual/hormonal dysfunction, and inconsistent long-term efficacy [2,3]. These limitations underscore the need for multi-target natural alternatives capable of simultaneously modulating the molecular pathways implicated in AGA pathogenesis.

Beyond direct androgen signaling, HFDPCs function as master regulators of follicular homeostasis through reciprocal paracrine crosstalk with adjacent cell populations in the perifollicular microenvironment, including dermal fibroblasts, keratinocytes, and vascular endothelial cells. HFDPC-secreted paracrine factors—principally VEGF and Wnt ligands—activate β-catenin-dependent transcriptional programs and promote angiogenesis in neighboring fibroblasts, collectively strengthening the perifollicular dermal niche and sustaining the vascular supply required for anagen maintenance [2]. Disruption of this HFDPC-fibroblast paracrine axis under conditions of androgen excess and oxidative stress therefore represents an underappreciated contributor to follicular regression in AGA, and its restoration constitutes a meaningful functional target for hair loss intervention. Furthermore, oxidative stress in AGA is not limited to ROS accumulation but extends to downstream lipid peroxidation, in which ROS react with polyunsaturated fatty acids in HFDPC membranes to generate malondialdehyde (MDA)—a stable biomarker of oxidative membrane damage that has been found at elevated levels in AGA patients relative to healthy controls. Lipid peroxidation-driven membrane disruption further compromises HFDPC proliferative capacity and paracrine signaling function, establishing a mechanistic link between oxidative stress and the progressive impairment of follicular regenerative activity that characterizes AGA pathogenesis [3,4].

Natural products, particularly coffee pulp from Arabica coffee (*Coffea arabica* L.), a major processing by-product, have gained substantial attention as rich sources of bioactive polyphenols, flavonoids, and caffeine with potent antioxidant and anti-inflammatory properties relevant to AGA pathogenesis [5]. However, the functional bioactivity of such raw extracts is often constrained by the limited bioavailability and structural complexity of their native phytochemical constituents. Microbial fermentation represents a powerful bioprocess strategy to overcome these limitations. By deploying microorganisms that produce key hydrolytic and biotransformation enzymes—including β-glucosidases, esterases, and phenolic acid decarboxylases—this process releases bound phenolics and converts them into more bioavailable, lower-molecular-weight bioactive forms [6,7]. Furthermore, fermentation concurrently generates an array of metabolites, including organic acids, bioactive peptides, exopolysaccharides, and microbial cell wall components. Collectively referred to as fermentation-derived metabolites, these molecules exert complementary antioxidant, anti-inflammatory, and immunomodulatory effects, providing a strong mechanistic rationale for the enhanced functional potential of fermented plant substrates compared to their non-fermented counterparts [7]. Beyond its bioactive potential, coffee pulp represents a substantial agro-industrial residue. Coffee-processing by-products, including coffee pulp, have traditionally been discarded or used in relatively low-value applications but have increasingly been explored for valorization as animal feed, biofuel feedstocks, biosorbents, enzyme-production substrates, and infusions [5]. Their bioactive constituents have also attracted interest for the development of functional ingredients in food, cosmetic, and pharmaceutical applications [5]. Nevertheless, untreated accumulation and uncontrolled decomposition of coffee pulp can contribute to environmental burden. Microbial fermentation therefore offers a value-added valorization route for this underutilized biomass, aligning with circular bioeconomy principles by converting a low-value processing residue into a functional cosmeceutical ingredient; mixed-culture fermentation, in particular, may further enhance this valorization potential by promoting microbial biotransformation and substrate utilization.

Among fermentative microorganisms, *Saccharomyces cerevisiae* and *Lactobacillus plantarum* are well-characterized species with complementary metabolic capabilities. *S. cerevisiae* contributes to the production of vitamins, antioxidants, and immunomodulatory polysaccharides such as β-glucans, while *L. plantarum* facilitates the conversion of complex phenolics into bioavailable forms and produces lactic acid and bioactive metabolites with established anti-inflammatory activity. Co-fermentation of these microorganisms has been shown to generate synergistic metabolic interactions, leading to enhanced release of phenolic compounds and the formation of diverse fermentation-derived metabolite profiles with improved biological activity [8,9,10,11].

Despite these advances, the effects of fermented coffee pulp on the molecular mechanisms underlying hair loss and hair growth have not been systematically investigated. In particular, it remains unclear how different fermentation strategies—single versus co-microbial cultures—influence the bioactive composition of coffee pulp and its capacity to modulate key in vitro mechanisms relevant to AGA, including SRD5A-mediated androgen metabolism, TGF-β1-driven follicular regression, lipopolysaccharide (LPS)-induced nitric oxide production, H_2_O_2_-induced intracellular ROS generation and lipid peroxidation, the effect of HFDPC-conditioned medium on dermal fibroblast proliferation, and the activation of Wnt/β-catenin, Sonic Hedgehog, and VEGF-mediated angiogenic pathways in HFDPCs.

Therefore, the present study evaluates, for the first time, the impact of single and co-microbial fermentation using *S. cerevisiae*, *L. plantarum*, and their combination on the bioactive profile and biological activities of coffee pulp extracts in the context of AGA management. Fermented extracts were characterized by untargeted metabolomic profiling and comprehensively assessed across a multi-target mechanistic framework encompassing androgen metabolism (*SRD5A1* and *SRD5A2*), TGF-β1-mediated follicular regression, oxidative stress (intracellular ROS and TBARS assay), inflammation (nitric oxide production), hair growth–related signaling (*CTNNB1*, *SHH*, *SMO*, *GLI1*, and *VEGF*), cell viability protection under pharmacological potassium-channel (K_ATP_) blockade, and the effect of HFDPC-conditioned medium on fibroblast proliferation using relevant in vitro cell models. By integrating fermentation strategy comparison with broad mechanistic coverage across both hair-loss and hair-growth axes, this work aims to provide the first mechanistic evidence supporting the potential of fermented coffee pulp as a potential source of bioactive ingredients for further cosmeceutical investigation.

## 2. Results

### 2.1. Total Phenolic Content and Antioxidant Properties of Coffee Pulp Extracts

#### 2.1.1. Total Phenolic Content of Coffee Pulp Extracts

The crude extracts of unfermented coffee pulp (CP) and microbially fermented coffee pulp (LP-CP: *Lactobacillus plantarum*-fermented coffee pulp; SC-CP: *Saccharomyces cerevisiae*-fermented coffee pulp; MIX-CP: mixed-culture fermented coffee pulp) were successfully obtained via ultrasonic-assisted extraction using 95% (*v*/*v*) ethanol as a solvent. Visual examination showed that all concentrated coffee pulp extracts were brownish liquids, with distinguishable variations in color intensity among the treatment groups, ranging from amber brown to dark brown. These observations were recorded as descriptive physical characteristics of the extracts. The potential compositional differences among the treatments were subsequently evaluated using phytochemical and metabolomic analyses. To further investigate these modifications, the specific bioactive profiles were evaluated.

**Figure 1:**
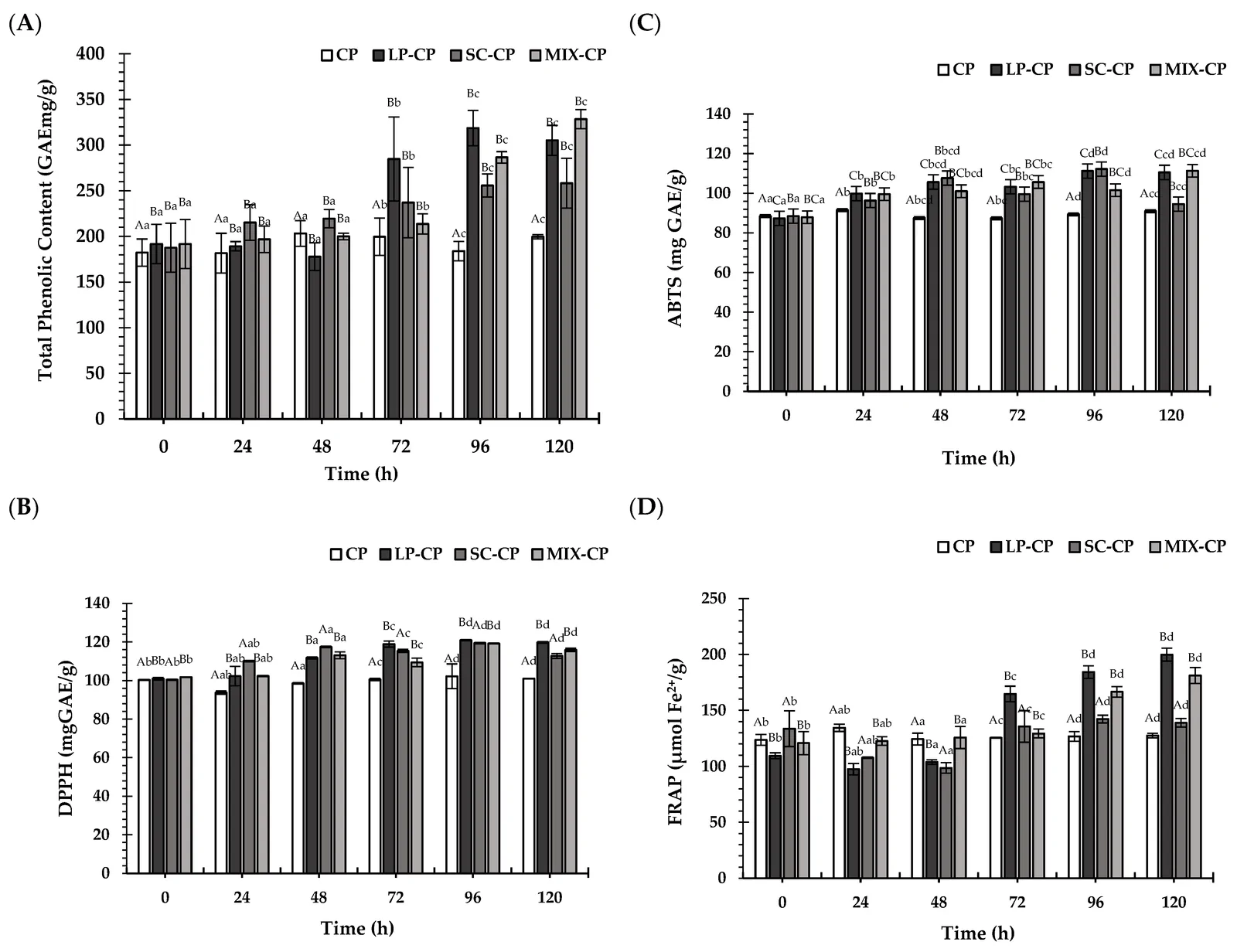
Effect of fermentation time and microbial strains on total phenolic content (TPC) (**A**), DPPH radical scavenging activity (**B**), ABTS radical scavenging activity (**C**), and Ferric reducing antioxidant power (FRAP) (**D**) of fermented coffee pulp extracts during fermentation at 28 °C. Capital letters represent significant differences between treatment groups at the same time point; lowercase letters denote significant differences within the same treatment group over time (*p* < 0.05). CP, unfermented coffee pulp; LP-CP, *Lactobacillus plantarum*-fermented coffee pulp; SC-CP, *Saccharomyces cerevisiae*-fermented coffee pulp; MIX-CP, mixed-culture fermented coffee pulp.

The total phenolic content (TPC) of fermented coffee pulp extracts by different microbial treatments showed distinct patterns over the 120 h fermentation period. The control group exhibited relatively stable TPC throughout fermentation, suggesting that spontaneous enzymatic or chemical transformations in the absence of inocula were minimal. The LP-CP treatment produced the most pronounced increase in TPC, followed by MIX-CP, with peak TPC values observed between 72 and 120 h. The TPC results demonstrated that all inoculated treatments produced significantly higher phenolic content than the control group throughout the entire fermentation period (Figure 1A).

#### 2.1.2. Antioxidant Properties of Coffee Pulp Extracts

DPPH radical scavenging activity (Figure 1B) followed a broadly similar trajectory to TPC across all treatment groups. All inoculated treatments exhibited markedly higher DPPH values than the control from the earliest time point measured (24 h), with values climbing during the initial phase of fermentation and declining or stabilizing at later time points.

The ABTS radical cation scavenging activity for all treatment groups is presented in Figure 1C. At the onset of fermentation (0 h), the control, SC-CP, LP-CP, and MIX-CP groups exhibited comparable values with no statistically significant differences, indicating a baseline antioxidant capacity inherent to the substrate. However, from 24 h onward, a clear differentiation emerged. The inoculated treatments showed a progressive increase in ABTS activity, peaking prominently at 48 h and again between 96 and 120 h, whereas the control remained significantly lower throughout the study. Furthermore, the LP-CP and MIX-CP groups achieved the highest overall ABTS values at the 96–120 h mark, demonstrating statistical superiority over both the SC-CP treatment and the control.

Within-group temporal variation revealed that ABTS activity did not increase monotonically but instead showed fluctuations across the 120 h. Notably, the control group exhibited its own temporal variation despite low absolute values, suggesting that even natural substrate chemistry undergoes minor changes over the fermentation duration at 28 °C, possibly due to limited activity of indigenous microflora or spontaneous oxidative processes. The inoculated groups showed more pronounced and earlier temporal shifts, reflecting the active metabolic contribution of the starter cultures. The fact that ABTS activity remained elevated in LP-CP and MIX-CP groups at 96–120 h, rather than declining sharply as observed in DPPH, may reflect the broader reactivity profile of the ABTS assay capturing sustained production of diverse radical-scavenging metabolites even as the most reactive hydrogen-donating phenolics begin to be consumed.

The FRAP analysis revealed a distinct and consistent separation between the inoculated treatments and the control group throughout the 120 h fermentation period (Figure 1D). A notable feature of these results was the broad equivalence in reductive capacity among the SC-CP, LP-CP, and MIX-CP groups at most time points.

### 2.2. Global Metabolomic Profiling and Multivariate Statistical Analysis of Fermented Coffee Pulp

A total of 2951 putatively annotated metabolite features were retained for downstream comparative analysis across the three fermentation conditions (LP-CP, SC-CP, and MIX-CP) relative to the non-fermented control. In the overall dataset without a statistical cutoff (Table 1), the proportion of metabolites showing increased abundance was highest in the co-culture (MIX-CP vs. CP) comparison (81.23%), followed by SC-CP vs. CP (62.92%) and LP-CP vs. CP (55.54%). Correspondingly, the proportion of decreased metabolites was lowest in the co-culture (18.77%), indicating a predominantly upward pattern of metabolite abundance under mixed-fermentation conditions.

When applying a stringent significance filter (adjusted *p*-value < 0.01 and fold change >2 or <0.5) (Table 2), the number of significantly altered metabolites decreased, but the same overall trend was observed. The co-culture (MIX-CP) comparison showed the highest proportion of significantly up-regulated metabolites (84.10%), followed by LP-CP vs. CP (65.29%) and SC-CP vs. CP (59.49%). Down-regulated metabolites accounted for 15.90%, 34.71%, and 40.51% in the co-culture, bacteria, and yeast comparisons, respectively.

Under the stringent statistical criteria, LP-CP vs. CP showed the largest number of significantly altered metabolite features (1020), comprising 666 upregulated and 354 downregulated features. In comparison, SC-CP vs. CP showed 158 significantly altered features (94 upregulated and 64 downregulated), whereas MIX-CP vs. CP showed 283 significantly altered features (238 upregulated and 45 downregulated). Although MIX-CP exhibited the highest proportion of upregulated features (84.10%) within its significantly altered metabolite set, this percentage reflects the direction of change rather than the overall extent of metabolic alteration. Thus, LP-CP produced the greatest number of statistically significant metabolic changes, whereas MIX-CP was characterized by a predominantly upward direction of the significant changes.

Inspection of the putative metabolite annotations indicated that the significantly altered features represented several broad structural classes. Up-abundance features included phenolic acids and flavonoid-related compounds, fatty acids and oxygenated lipid derivatives, organic acids, and amino-acid-related metabolites. The down-abundance features were likewise chemically diverse. In LP-CP, representative decreased features included phenolic compounds and glycosides, such as gallic acid, apigenin, and gaultherin, together with glycerophospholipid species, including phosphatidylcholine (PC), phosphatidylethanolamine (PE), phosphatidylinositol (PI), and phosphatidic acid (PA), as well as several oxygenated fatty-acid and oxylipin-related features. In SC-CP and MIX-CP, decreased features similarly included phenolic acids and flavonoid-related metabolites, such as gallic acid, syringic acid, isorhamnetin, Icariside F2, and quercetin glycosides, together with phospholipid-, ceramide-, and epoxy/hydroxy-fatty-acid-related features. These structural assignments are based on putative annotations from untargeted LC–MS/MS analysis and should therefore not be interpreted as definitive structural identifications.

**Figure 2:**
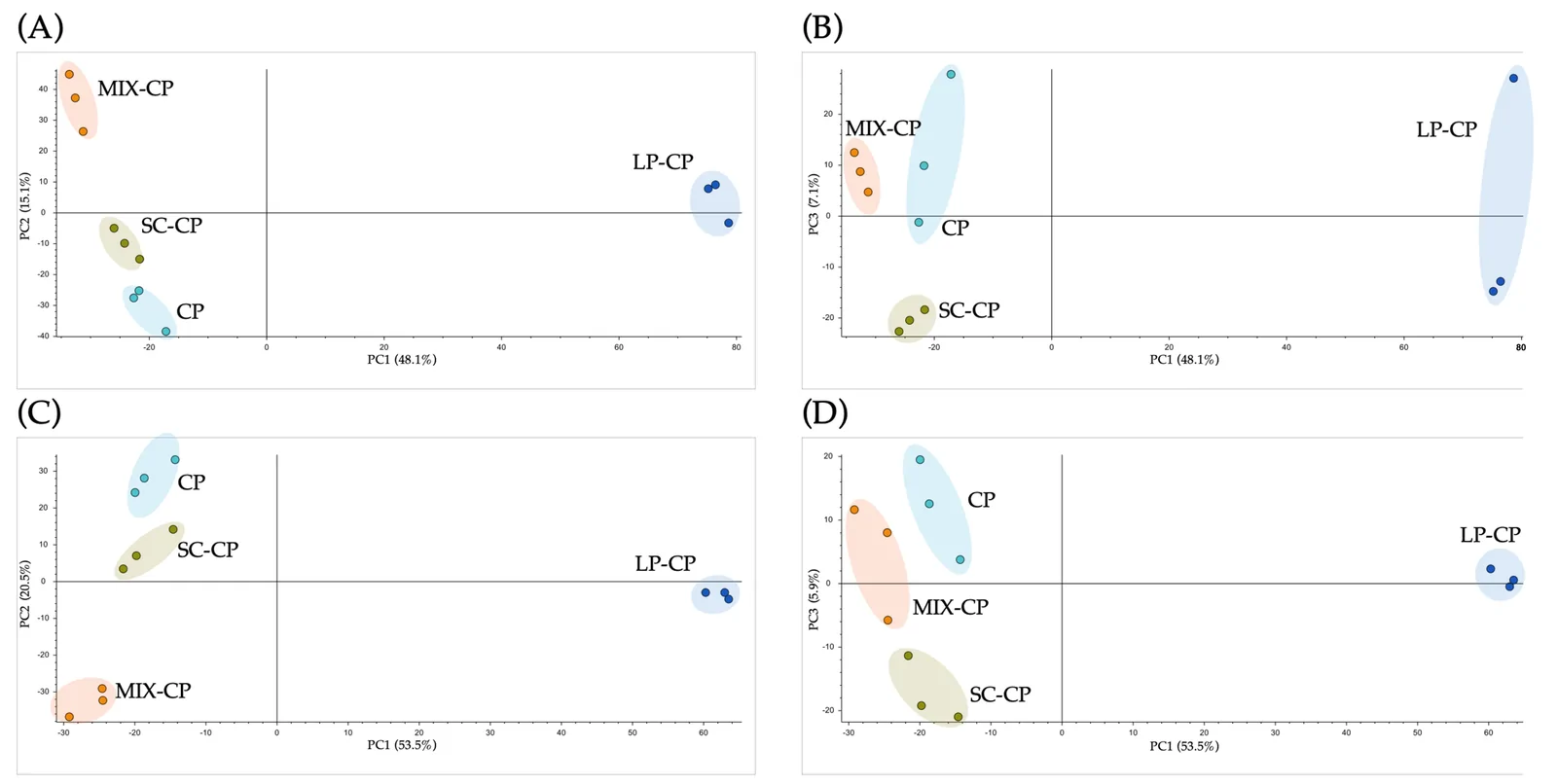
PCA of metabolomic profiles of coffee pulp extracts under different fermentation conditions. Score plots showing sample distribution in positive ion mode, (**A**) PC1 vs. PC2 and (**B**) PC1 vs. PC3, and negative ion mode, (**C**) PC1 vs. PC2 and (**D**) PC1 vs. PC3. Each point represents an individual replicate. CP, unfermented coffee pulp; LP-CP, *Lactobacillus plantarum*-fermented coffee pulp; SC-CP, *Saccharomyces cerevisiae*-fermented coffee pulp; MIX-CP, mixed-culture fermented coffee pulp.

Principal component analysis (PCA) was performed to evaluate global metabolic variation among the control, bacterial fermentation, yeast fermentation, and co-culture fermentation samples (Figure 2). The PCA score plots revealed distinct clustering among the four treatment groups, indicating fermentation-strategy-dependent differences in the metabolomic profiles.

The PCA score plots revealed distinct treatment-dependent clustering of the metabolomic profiles in both positive and negative ionization modes. Among the fermented treatments, LP-CP showed the most pronounced separation from the other groups along PC1, indicating that fermentation with *L. plantarum* accounted for a substantial component of the overall metabolomic variation. In contrast, SC-CP and MIX-CP occupied distinct positions relative to CP and LP-CP across the additional principal components (PC2 and PC3), suggesting that the different fermentation strategies generated characteristic patterns of metabolic remodeling rather than a uniformly greater shift in MIX-CP. The close clustering of biological replicates within each treatment group indicated good analytical reproducibility.

### 2.3. Polyphenol Profile of Fermented Coffee Pulp

The polyphenol profile of unfermented and fermented coffee pulp extracts is summarized in Table 3. Among the fermented extracts, LP-CP contained the highest levels of polyphenol compounds, including gallic acid, catechin, epicatechin, and *o*-coumaric acid, with epicatechin reaching 76.02 ± 0.36 mg/g extract, markedly higher than in MIX-CP, CP, and SC-CP.

Despite LP-CP exhibiting the highest abundance of several individual polyphenol compounds, MIX-CP displayed a more favorable bioactive polyphenol profile, with the highest contents of rosmarinic acid (3.22 ± 0.05 mg/g extract) and quercetin (21.75 ± 0.23 mg/g extract).

### 2.4. Cytotoxic Assessment and Proliferative Response of Cells Treated with Fermented Coffee Pulp Extracts

The cytotoxic potential of ultrasonic-assisted ethanol extracts from unfermented coffee pulp (CP) and fermented coffee pulp at the 120 h fermentation period including, *Lactobacillus plantarum*-fermented coffee pulp (LP-CP), *Saccharomyces cerevisiae*-fermented coffee pulp (SC-CP), and mixed-culture fermented coffee pulp (MIX-CP) was evaluated across ten two-fold serial dilutions spanning 2.000–0.004 mg/mL in four cell lines: HFDPC, murine macrophages (RAW 264.7), hTERT-immortalized fibroblasts (hTERT), and human prostate cancer (DU-145) cells. The four cell types were selected based on their distinct and complementary biological functions, with each model addressing a specific experimental objective. Although DU-145 cells are derived from prostatic tissue rather than hair follicles, their substantial basal expression of 5α-reductase provides an independent model for corroborating androgen-metabolism–related observations obtained in HFDPCs. HFDPCs served as the principal follicular model because they constitute a major functional cell population within the hair bulb, where their proliferative activity contributes to hair-follicle cycling and hair growth. RAW 264.7 cells provided a complementary inflammation model because of their established and reproducible response to lipopolysaccharide stimulation, supporting the interpretation of inflammatory effects observed in HFDPCs. Finally, hTERT fibroblasts served as responder cells in conditioned-medium assays, allowing the assessment of indirect biological effects potentially mediated by soluble factors released from HFDPCs within the follicular microenvironment [12].

In accordance with ISO 10993-5 [13], concentrations sustaining cell viability above 80% relative to untreated controls were designated as non-cytotoxic. Overall, RAW 264.7, hTERT, and DU-145 cells tolerated all extracts up to 0.250 mg/mL without significant reductions in viability, whereas HFDPC viability was maintained only at concentrations up to 0.125 mg/mL. As HFDPCs represented the most sensitive cell type, 0.125 mg/mL was adopted as the highest common non-cytotoxic concentration for all subsequent cell-based experiments [14]. Complete viability data across all concentrations and cell lines are provided in Supplementary Table S1.

Beyond the cytotoxicity threshold, the proliferative response of HFDPCs to sub-cytotoxic concentrations of fermented coffee pulp extracts was further assessed. Notably, MIX-CP stimulated HFDPC proliferation to 146.38 ± 2.98% at 0.016 mg/mL, significantly exceeding the untreated control (*p* < 0.05) and remaining markedly higher than the monoculture-fermented extracts (*p* < 0.05)—specifically LP-CP 104.12 ± 1.83%, SC-CP 102.52 ± 3.22%, and CP 101.07 ± 2.65%, respectively, all of which remained near basal proliferative levels at the same concentration. This selective pro-proliferative effect of MIX-CP at low concentrations occurred alongside a distinct metabolomic profile generated by mixed-culture fermentation. Although MIX-CP showed fewer significantly altered features than LP-CP, most of the altered features in MIX-CP exhibited increased abundance relative to CP. Thus, the enhanced proliferative response observed here is unlikely to reflect a greater overall extent of metabolomic remodeling and may instead be related to qualitative differences in the metabolite composition generated under mixed-culture fermentation. However, the specific metabolites responsible for this response were not identified in the present study.

### 2.5. Effect of HFDPC-Conditioned Medium from Fermented Coffee Pulp Extracts on Human Fibroblast Proliferation

**Figure 3:**
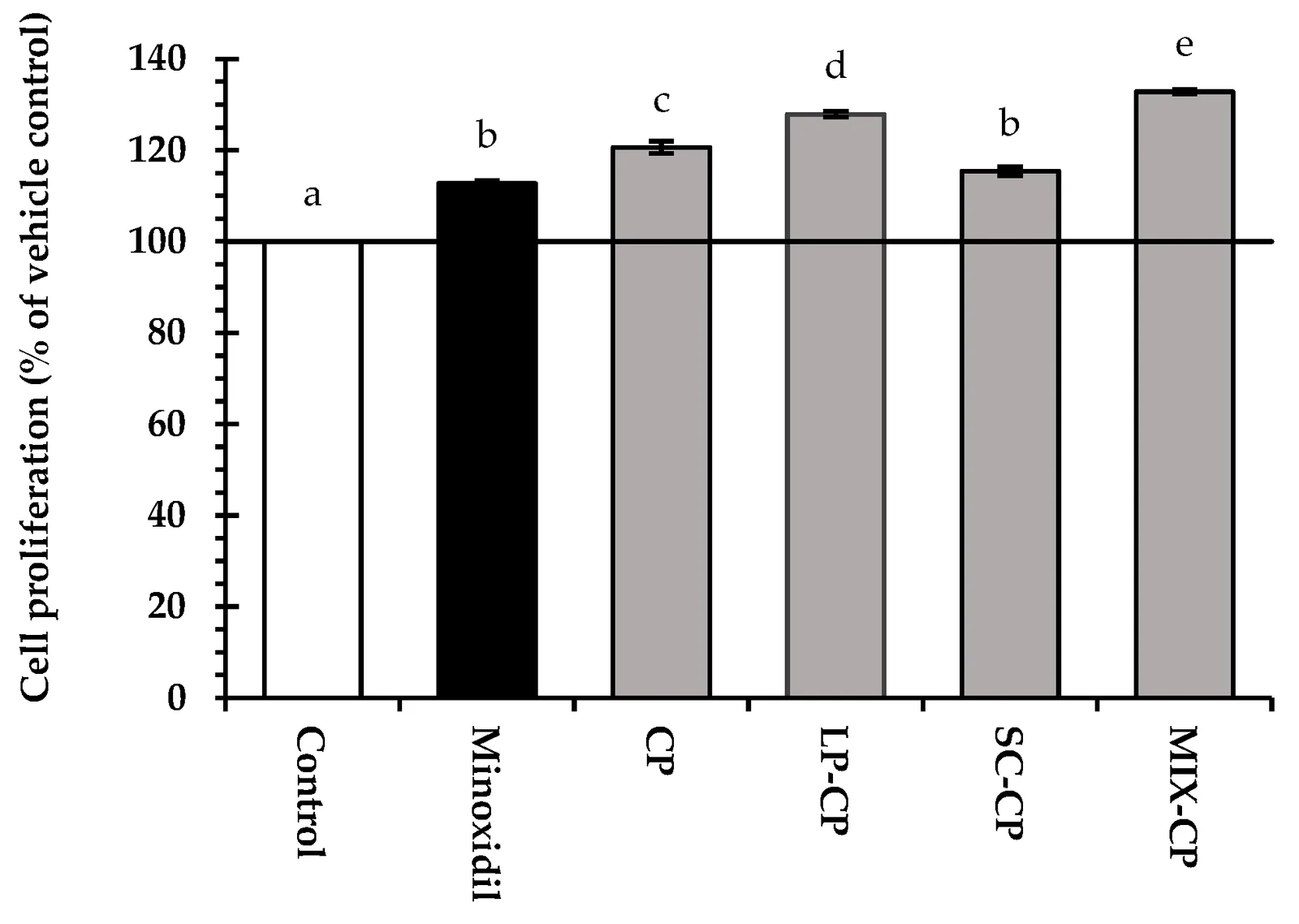
Proliferative response of human fibroblasts cultured in conditioned medium harvested from HFDPCs pre-treated with fermented and unfermented coffee pulp extracts at 0.125 mg/mL, relative to the vehicle control (culture medium-treated HFDPCs) and a standard treatment (minoxidil). Values are expressed as mean ± SD (*n* = 3). One-way ANOVA followed by Tukey’s HSD post hoc test was applied; non-overlapping letters (a–e) indicate statistically significant differences among groups (*p* < 0.05). CP: unfermented coffee pulp; LP-CP: *Lactobacillus plantarum*-fermented coffee pulp; SC-CP: *Saccharomyces cerevisiae*-fermented coffee pulp; MIX-CP: mixed-culture fermented coffee pulp.

The effect of conditioned medium collected from extract-treated HFDPC cultures on human fibroblast proliferation was evaluated. Among all treatment groups, MIX-CP-conditioned medium induced the highest fibroblast proliferation at 132.84 ± 0.46% of the vehicle control, followed by LP-CP (127.89 ± 0.65%), CP (120.63 ± 1.36%), and SC-CP (115.43 ± 1.02%), respectively (Figure 3). MIX-CP, LP-CP, and CP-conditioned media produced significantly greater fibroblast proliferative responses than the minoxidil-conditioned medium (112.74 ± 0.65%), whereas the response to SC-CP-conditioned medium was numerically higher but not significantly different from that of the minoxidil-conditioned medium.

The observed differences in fibroblast proliferative response among the treatment groups occurred alongside distinct fermentation-dependent metabolite profiles. As with the direct HFDPC proliferation response described in Section 2.4, MIX-CP showed fewer significantly altered features than LP-CP, despite a predominantly upward pattern of change (Section 2.2). Thus, the greater fibroblast proliferative response induced by MIX-CP-conditioned medium is unlikely to be explained simply by a broader extent of metabolomic remodeling and may instead reflect qualitative differences in the metabolite composition generated under mixed-culture fermentation. Because residual extract constituents were not completely excluded during conditioned-medium preparation, the relative contributions of direct extract effects and HFDPC-derived paracrine factors remain unresolved [12].

### 2.6. Protective Effects of Fermented Coffee Pulp Extracts Against Tolbutamide-Induced Reduction in HFDPC Viability

**Figure 4:**
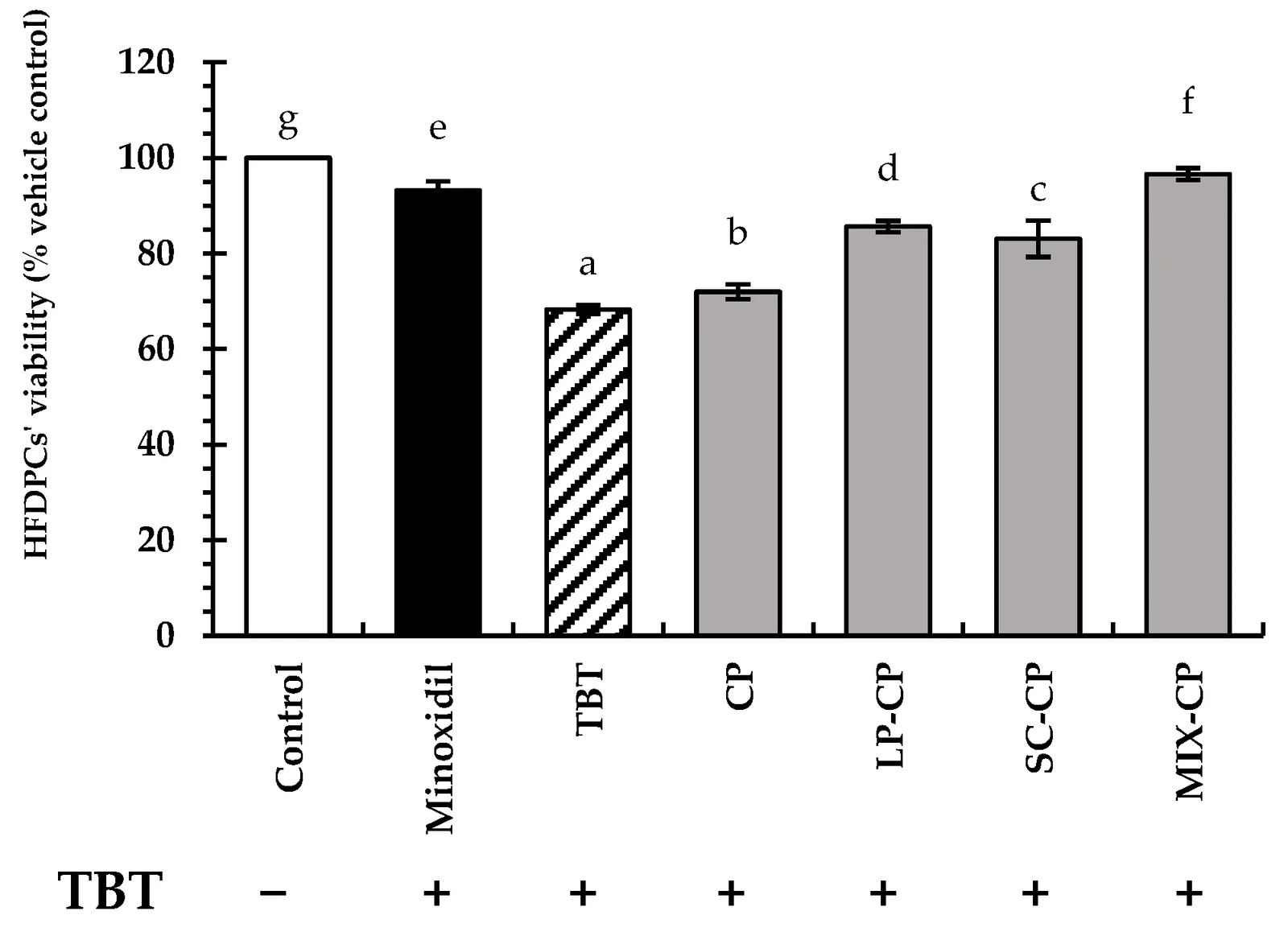
Effects of fermented and unfermented coffee pulp extracts (0.125 mg/mL) on HFDPC viability following exposure to tolbutamide, compared with the negative control (tolbutamide-treated cells) and standard treatment (minoxidil). Values are expressed as mean ± SD (*n* = 3). One-way ANOVA followed by Tukey’s HSD post hoc test was applied; groups sharing no common letter (a–g) differ significantly (*p* < 0.05). TBT: tolbutamide; CP: unfermented coffee pulp; LP-CP: *Lactobacillus plantarum*-fermented coffee pulp; SC-CP: *Saccharomyces cerevisiae*-fermented coffee pulp; MIX-CP: mixed-culture fermented coffee pulp.

The ability of fermented coffee pulp extracts to attenuate tolbutamide-induced reduction in HFDPC viability was evaluated using a tolbutamide (TBT)-based inhibition model. TBT treatment alone (negative control) reduced HFDPC viability to 68.25 ± 0.95% relative to the untreated control (Figure 4). Among all treatment groups, MIX-CP demonstrated the most pronounced viability-protective activity, elevating cell viability to 96.61 ± 1.24%—a level approaching that of the untreated control and numerically exceeding minoxidil (93.17 ± 1.95%). Furthermore, LP-CP, SC-CP, and non-fermented CP also significantly restored HFDPC viability compared to the TBT-treated group. These findings demonstrate that fermented coffee pulp extracts can effectively protect HFDPC viability against TBT-induced viability reduction.

### 2.7. Anti-Inflammatory Properties of Fermented Coffee Pulp Extracts in Cell-Based Models

**Figure 5:**
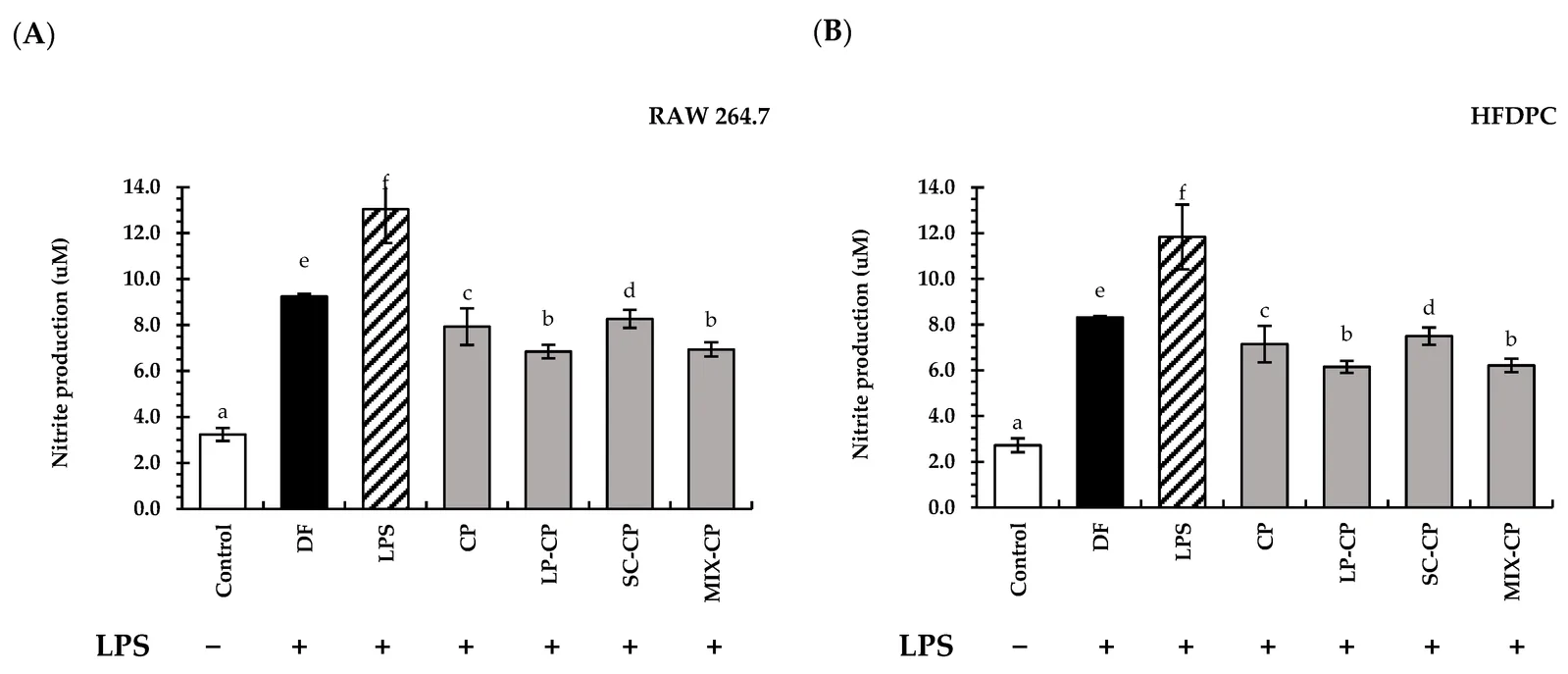
Inhibition of nitric oxide (NO) production in lipopolysaccharide (LPS)-stimulated (**A**) RAW 264.7 macrophage cells and (**B**) HFDPC cells by fermented and unfermented coffee pulp extracts at 0.125 mg/mL. Results were benchmarked against an untreated control, an LPS-stimulated negative control (without pre-treatment), and a reference anti-inflammatory agent (DF: diclofenac sodium). Data represent mean ± SD (*n* = 3); one-way ANOVA with Tukey’s HSD post hoc test was used for between-group comparisons. Non-overlapping letters (a–f) indicate statistically significant differences (*p* < 0.05). CP: unfermented coffee pulp; LP-CP: *Lactobacillus plantarum*-fermented coffee pulp; SC-CP: *Saccharomyces cerevisiae*-fermented coffee pulp; MIX-CP: mixed-culture fermented coffee pulp.

Inducible nitric oxide synthase (iNOS) is upregulated under conditions of oxidative stress, androgen exposure, and inflammatory signaling, prompting perifollicular macrophages to release excessive nitric oxide (NO), which perpetuates follicular inflammation and accelerates hair follicle miniaturization. Elevated NO levels in HFDPCs have been documented in patients with androgenetic alopecia, implicating NO-mediated inflammatory cascades as a key contributor to pathological hair loss [15]. In the present study, LPS stimulation significantly elevated nitrite accumulation in both HFDPCs (11.83 ± 1.42 µM) and RAW 264.7 macrophages (13.04 ± 1.47 µM) relative to untreated controls, confirming successful inflammatory induction (Figure 5). Pre-treatment with all coffee pulp extracts substantially reduced nitrite output in both cell types compared with the LPS-stimulated negative control (*p* < 0.05).

Among the coffee pulp extracts, LP-CP exhibited the greatest NO-suppressing activity in both HFDPCs (6.15 ± 0.27 µM) and RAW 264.7 macrophages (6.84 ± 0.30 µM), followed closely by MIX-CP (6.21 ± 0.29 and 6.94 ± 0.31 µM, respectively), with no statistically significant difference observed between these two groups (*p* > 0.05). Notably, this trend directly aligns with the previously described bioactive profiles, where the TPC and primary antioxidant capacities of LP-CP and MIX-CP were similarly non-significantly different. Both treatments significantly outperformed diclofenac sodium (8.31 ± 0.06 and 9.24 ± 0.12 µM). Furthermore, SC-CP and CP also reduced NO production relative to the LPS control, albeit with lesser magnitude.

### 2.8. Suppression of Intracellular Reactive Oxygen Species by Fermented Coffee Pulp Extracts

**Figure 6:**
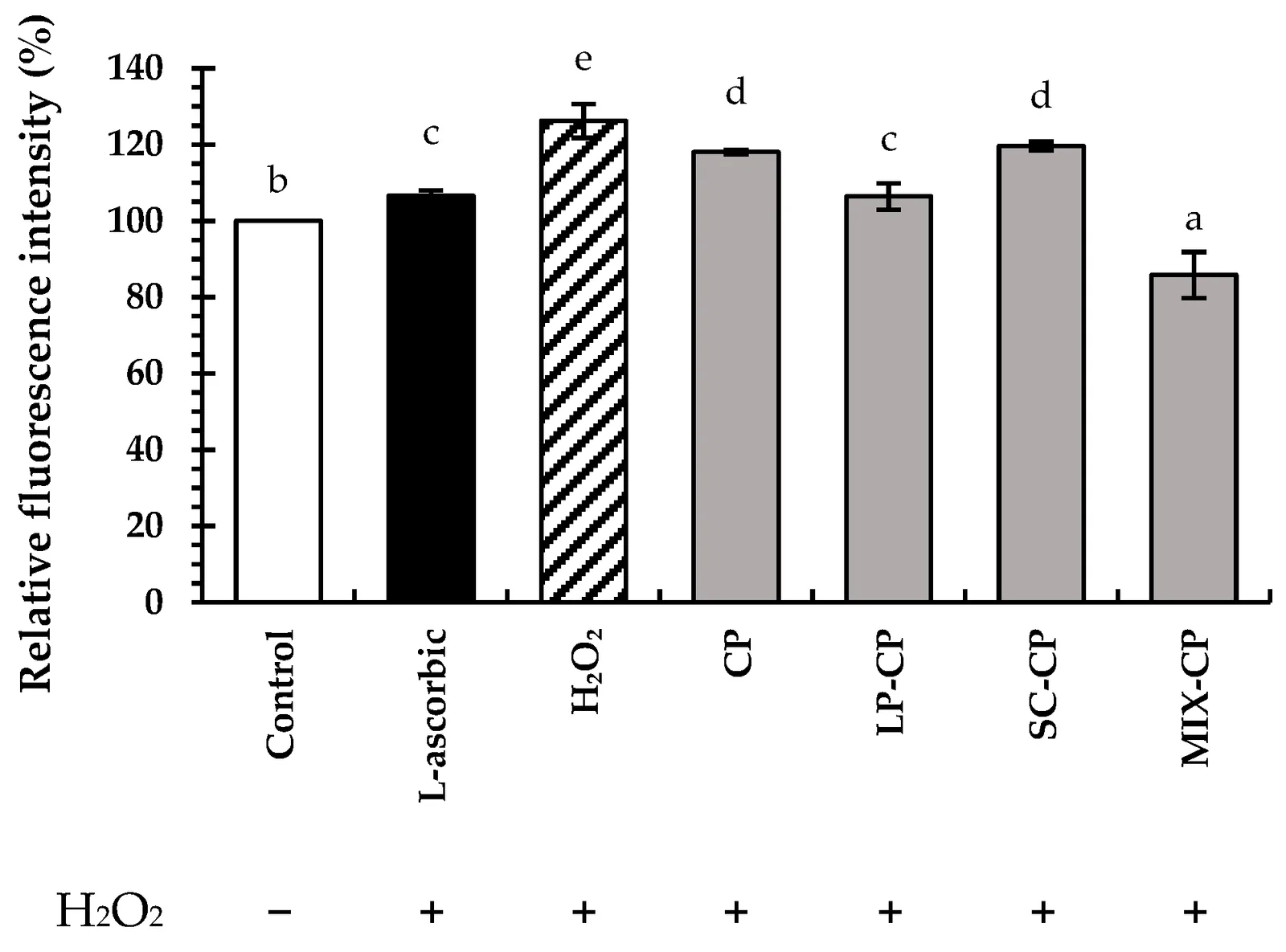
Intracellular ROS levels in hydrogen peroxide (H_2_O_2_)-challenged HFDPCs following pre-treatment with fermented and unfermented coffee pulp extracts at 0.125 mg/mL, benchmarked against the untreated control, the H_2_O_2_-induced negative control (without pre-treatment), and L-ascorbic acid as a reference antioxidant standard. Values are expressed as mean ± SD (*n* = 3). One-way ANOVA followed by Tukey’s HSD post hoc test was applied; non-overlapping letters (a–e) indicate statistically significant differences among groups (*p* < 0.05). CP: unfermented coffee pulp; LP-CP: *Lactobacillus plantarum*-fermented coffee pulp; SC-CP: *Saccharomyces cerevisiae*-fermented coffee pulp; MIX-CP: mixed-culture fermented coffee pulp.

Oxidative stress is a shared pathophysiological feature across multiple forms of alopecia, with excess reactive oxygen species (ROS) impairing HFDPC proliferative capacity, inducing TGF-β-mediated follicular regression, and accelerating premature anagen-to-catagen transition [16]. Intracellular ROS levels were quantified in hydrogen peroxide (H_2_O_2_)-challenged HFDPCs using the H_2_DCFDA fluorescent probe to evaluate the capacity of coffee pulp extracts to attenuate oxidative burden at the cellular level. H_2_O_2_ exposure elevated relative fluorescence intensity to 126.21 ± 4.46% relative to the untreated control, confirming effective oxidative induction, while L-ascorbic acid, the reference antioxidant standard, partially attenuated this elevation (106.59 ± 1.44%) (Figure 6).

Among the coffee pulp extracts, MIX-CP exhibited the greatest intracellular ROS-lowering effect, with fluorescence intensity decreasing to 85.81 ± 6.06%, a level even below that of untreated cells. LP-CP also markedly reduced ROS generation to near-baseline levels (106.41 ± 3.48%), showing no significant difference from L-ascorbic acid, the positive control. Although SC-CP (119.63 ± 1.15%) and CP (118.06 ± 0.63%) were less effective than MIX-CP and LP-CP, both treatments nevertheless significantly attenuated intracellular ROS accumulation compared with the H_2_O_2_-treated control group.

### 2.9. Suppression of Lipid Peroxidation in H_2_O_2_-Challenged HFDPCs by Fermented Coffee Pulp Extracts

**Figure 7:**
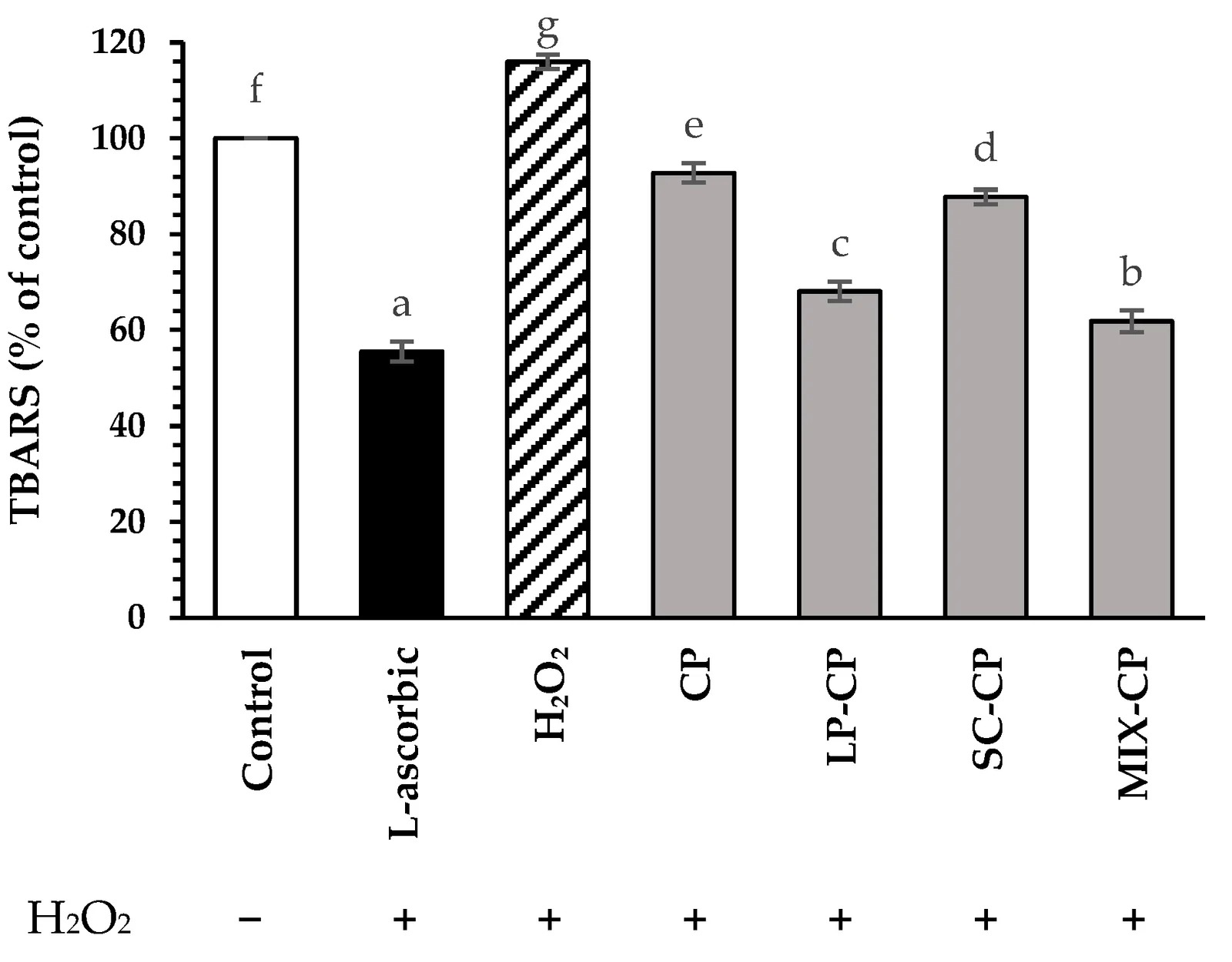
Attenuation of lipid peroxidation, assessed by TBARS levels, in H_2_O_2_-challenged HFDPCs following pre-treatment with fermented and unfermented coffee pulp extracts at 0.125 mg/mL. Comparisons were made against an untreated control, an H_2_O_2_-induced negative control (without pre-treatment), and a reference antioxidant standard. Results are expressed as mean ± SD (*n* = 3). Statistical significance was determined by one-way ANOVA with Tukey’s HSD post hoc test; groups marked with non-identical letters (a–g) are significantly different (*p* < 0.05). CP: unfermented coffee pulp; LP-CP: *Lactobacillus plantarum*-fermented coffee pulp; SC-CP: *Saccharomyces cerevisiae*-fermented coffee pulp; MIX-CP: mixed-culture fermented coffee pulp.

Lipid peroxidation represents a critical downstream consequence of oxidative stress, in which ROS react with polyunsaturated fatty acids in cellular membranes to generate malondialdehyde (MDA), a stable end-product of lipid peroxidation and a widely used biomarker of intracellular oxidative damage. Elevated TBARS levels have been reported in AGA patients relative to healthy controls, implicating lipid peroxidation-mediated cellular injury as a contributor to HFDPC dysfunction and follicular regression [17]. In the present study, H_2_O_2_ challenge significantly elevated TBARS levels in HFDPCs to 115.94 ± 1.53% of the untreated control, confirming effective oxidative induction, while L-ascorbic acid, the reference antioxidant standard, substantially attenuated MDA accumulation to 55.51 ± 2.08% of control (Figure 7).

Among the coffee pulp extracts, MIX-CP demonstrated the greatest suppression of lipid peroxidation, reducing TBARS levels to 61.84 ± 2.31% of control—approaching the efficacy of L-ascorbic acid and significantly outperforming all monoculture-fermented and unfermented treatments. LP-CP followed with a TBARS value of 68.08 ± 2.00%, while SC-CP (87.75 ± 1.53%) and unfermented CP (92.78 ± 2.02%) exhibited progressively lesser suppression, although all treatments significantly reduced MDA levels relative to the H_2_O_2_-induced control.

### 2.10. Transcriptional Modulation of Hair Loss-Related Genes in the Androgen and TGF-β Signalling Pathways by Fermented Coffee Pulp Extracts

AGA is mechanistically driven by the overproduction of DHT, a potent androgen synthesised from testosterone through the catalytic activity of SRD5A isoenzymes. Three SRD5A isoforms—SRD5A1, SRD5A2, and SRD5A3—are expressed in hair follicles, with SRD5A1 and SRD5A2 representing the primary therapeutic targets in AGA management [18]. Elevated DHT in androgen-sensitive follicular regions accelerates the transition from the anagen to the catagen phase, inducing progressive follicular miniaturisation and eventual hair loss [18]. DHT further perpetuates follicular regression by stimulating TGF-β1 expression in HFDPCs, which subsequently suppresses epithelial cell proliferation and drives apoptotic signaling, consolidating the premature anagen-to-catagen transition [19]. Pharmacological inhibition of SRD5A isoenzymes—the mechanism of action of dutasteride and finasteride—and modulation of downstream TGF-β1 signaling therefore constitute validated molecular strategies for interrupting AGA pathogenesis.

**Figure 8:**
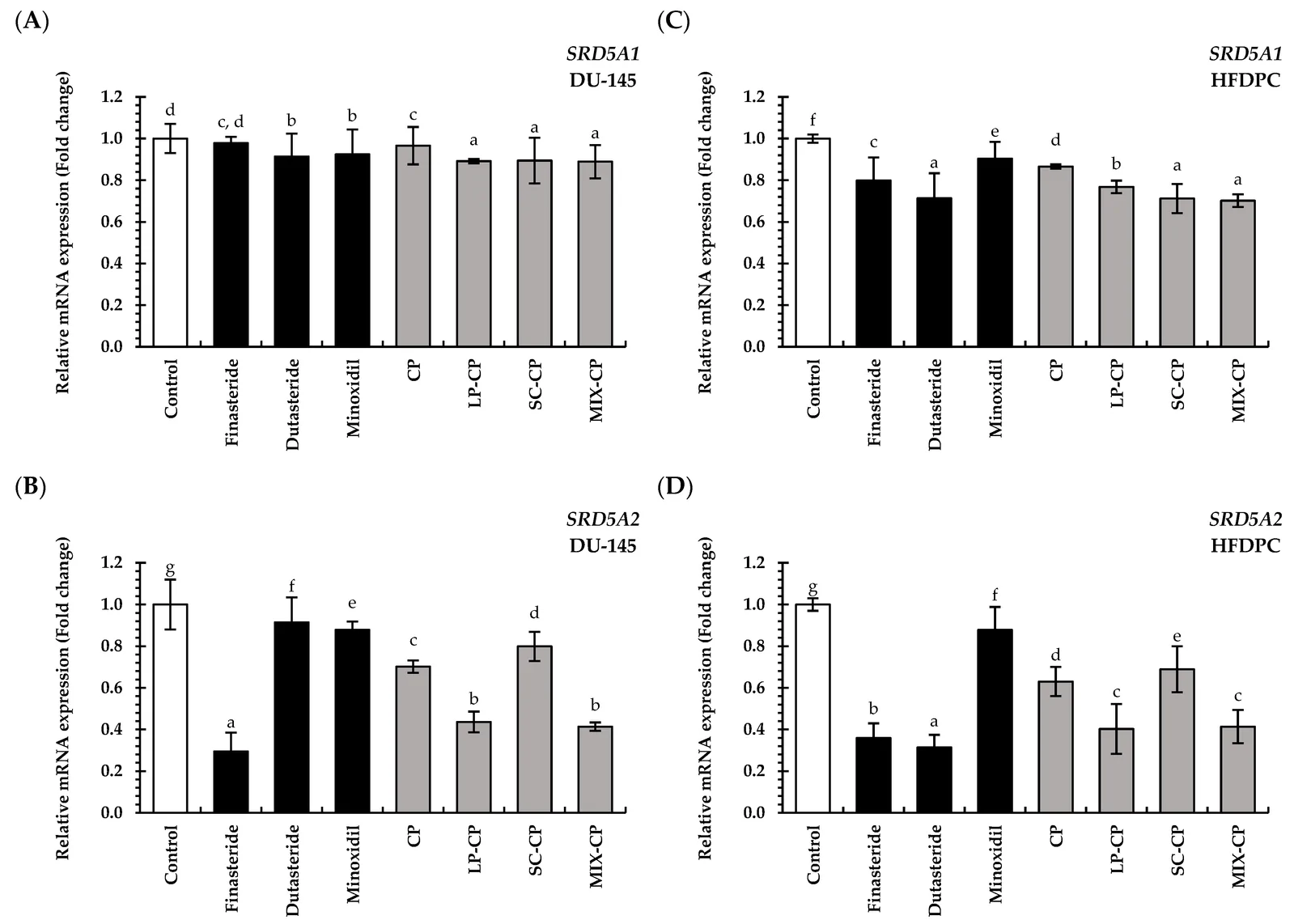
Relative mRNA expression of androgen-associated genes—(**A**) *SRD5A1* and (**B**) *SRD5A2*—in DU-145 cells and (**C**) *SRD5A1* and (**D**) *SRD5A2* in HFDPC cells, following treatment with fermented and unfermented coffee pulp extracts at 0.125 mg/mL. Expression levels were normalized to *GAPDH* and compared against relevant pharmacological standards (dutasteride, finasteride, and minoxidil). Data are presented as mean ± SD (*n* = 3); one-way ANOVA with Tukey’s HSD post hoc test was applied. Non-identical letters (a–g) indicate statistically significant between-group differences (*p* < 0.05). CP: unfermented coffee pulp; LP-CP: *Lactobacillus plantarum*-fermented coffee pulp; SC-CP: *Saccharomyces cerevisiae*-fermented coffee pulp; MIX-CP: mixed-culture fermented coffee pulp.

In the present study, all coffee pulp extracts significantly downregulated *SRD5A1* and *SRD5A2* mRNA expression in both DU-145 and HFDPC cells relative to the untreated control (*p* < 0.05) (Figure 8). Among the fermented treatments, MIX-CP demonstrated the most pronounced suppression across both cell types and both gene targets, with *SRD5A1* fold changes of 0.89 ± 0.08 in DU-145 and 0.70 ± 0.03 in HFDPCs, and *SRD5A2* fold changes of 0.41 ± 0.02 in DU-145 and 0.41 ± 0.08 in HFDPCs. LP-CP produced comparable suppression of *SRD5A2* (DU-145: 0.44 ± 0.05; HFDPCs: 0.40 ± 0.12), with no statistically significant difference from MIX-CP for this gene target (*p* > 0.05), consistent with the pattern observed across multiple bioactivity endpoints in this study where the *L. plantarum*-derived phenolic fraction contributes substantially to shared efficacy between LP-CP and MIX-CP. SC-CP exhibited intermediate activity, while unfermented CP showed the least suppression among all treatments.

Critically, both MIX-CP and LP-CP demonstrated significantly greater *SRD5A2* suppression than finasteride and minoxidil, a standard drug for hair loss treatment, in both cell types (*p* < 0.05), corresponding to approximately 1.5- and 2-fold greater suppressive effects on mRNA expression, respectively. For *SRD5A1*, all fermented extracts exhibited suppression levels comparable to or greater than those of dutasteride, finasteride, and minoxidil in both cell types.

**Figure 9:**
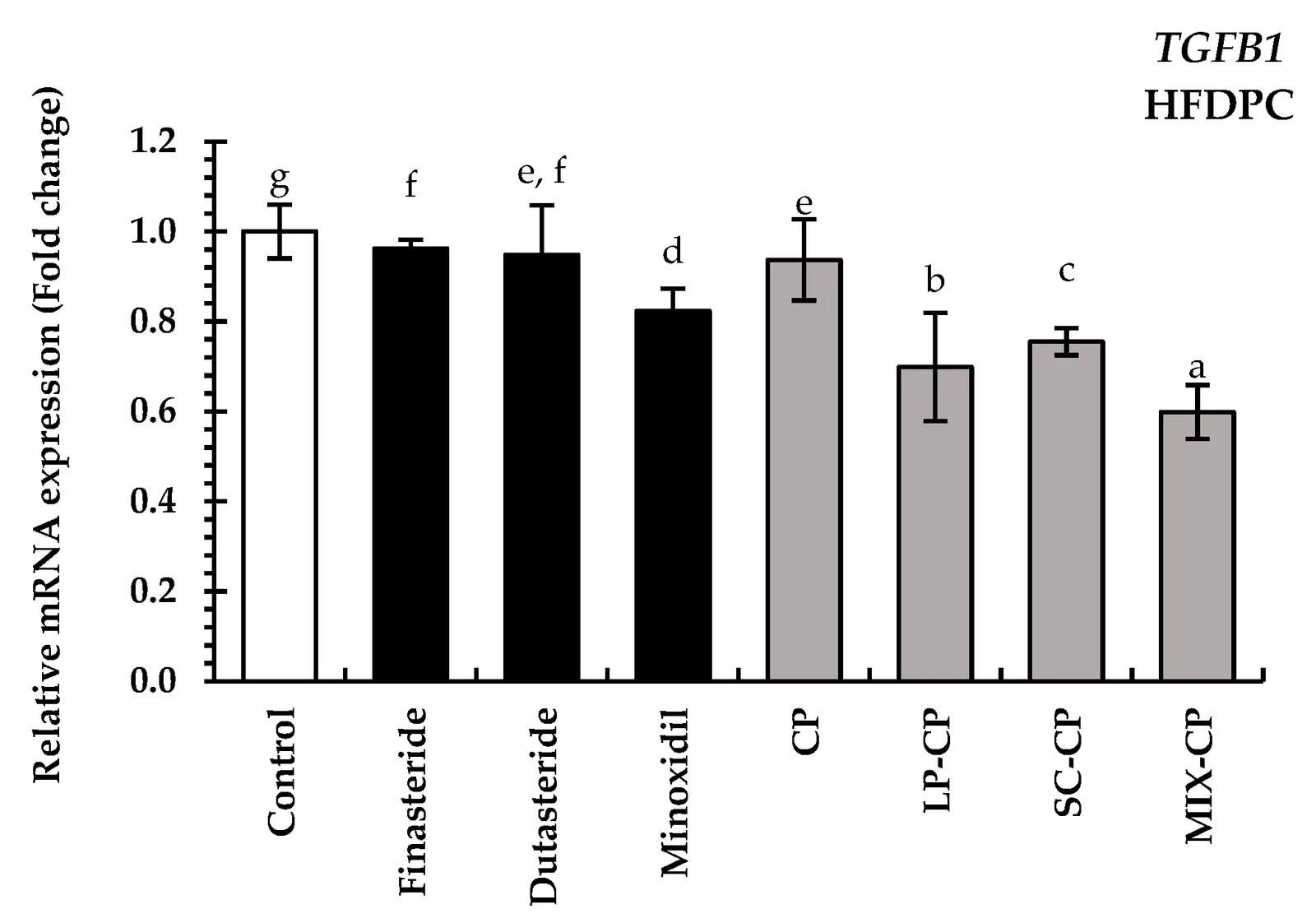
Relative mRNA expression of *TGFB1*, a key mediator of the transforming growth factor-beta (TGF-β) signaling pathway, in HFDPCs following treatment with fermented and unfermented coffee pulp extracts at 0.125 mg/mL, benchmarked against pharmacological reference standards. All expression levels were normalized to *GAPDH* as an internal reference. Data are presented as mean ± SD (*n* = 3). Statistical analysis was performed using one-way ANOVA with Tukey’s HSD post hoc test; non-overlapping letters (a–g) denote statistically significant differences between groups (*p* < 0.05). CP: unfermented coffee pulp; LP-CP: *Lactobacillus plantarum*-fermented coffee pulp; SC-CP: *Saccharomyces cerevisiae*-fermented coffee pulp; MIX-CP: mixed-culture fermented coffee pulp.

Regarding the downstream androgen signaling axis, all fermented extracts exhibited TGFB1 suppression levels that were significantly greater than all reference standards (dutasteride, finasteride, and minoxidil) (Figure 9). In particular, MIX-CP (0.60 ± 0.06) provided the greatest suppression of *TGFB1* among the fermented extracts, followed by LP-CP (0.70 ± 0.12), SC-CP (0.75 ± 0.03), and CP (0.94 ± 0.09), respectively.

### 2.11. Transcriptional Modulation of Hair Growth-Related Genes via Wnt/β-Catenin, Sonic Hedgehog, and Angiogenic Signaling Pathways by Fermented Coffee Pulp Extracts

Hair follicle morphogenesis and the maintenance of the anagen growth phase are coordinately governed by the Wnt/β-catenin and Sonic Hedgehog signaling cascades, which function as sequential and interdependent regulatory axes in HFDPCs. In the canonical Wnt/β-catenin pathway, Wnt ligands engage Frizzled receptors and co-receptors to stabilise cytoplasmic β-catenin, preventing its proteasomal degradation and enabling its nuclear translocation to activate *CTNNB1*-dependent transcription of genes that promote HFDPC proliferation, migration, and the induction of follicular anagen. The Wnt/β-catenin pathway acts as an upstream activator of the Sonic Hedgehog signaling cascade, in which SHH ligands bind to the Patched (PTCH) receptor, relieving repression of the Smoothened (SMO) transmembrane protein. Activated SMO translocates to the primary cilium and recruits glioma-associated oncogene (GLI) transcription factors to form a macromolecular complex that translocates to the nucleus and drives the transcription of downstream target genes, collectively promoting the telogen-to-anagen transition and sustaining HFDPC proliferative activity during the growth phase [2].

**Figure 10:**
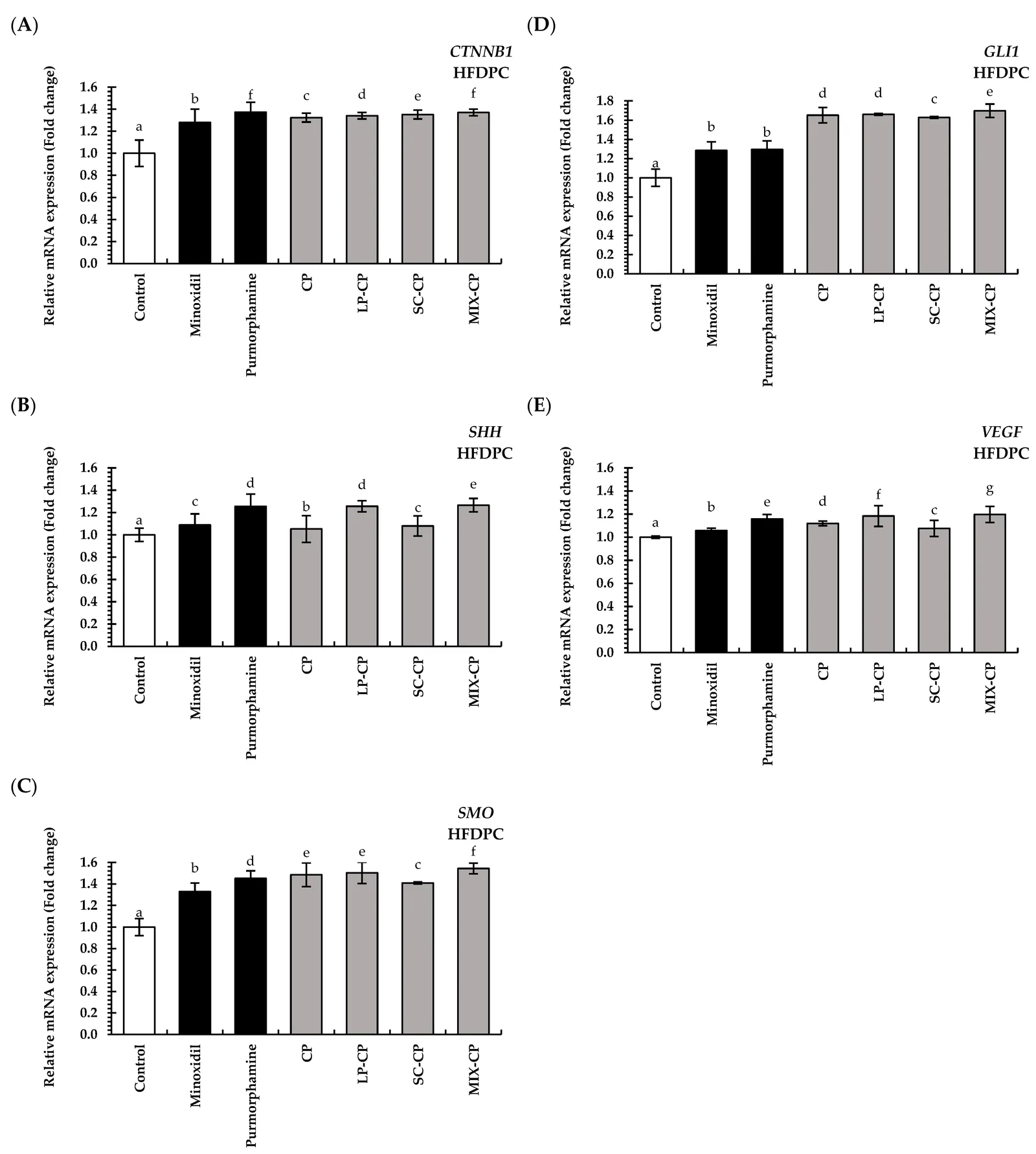
Transcriptional modulation of hair regeneration-related genes in HFDPC cells by fermented and unfermented coffee pulp extracts (0.125 mg/mL), including components of the Wnt/β-catenin pathway: (**A**) *CTNNB1*; the Sonic Hedgehog pathway: (**B**) *SHH*, (**C**) *SMO*, and (**D**) *GLI1*; and the angiogenic factor (**E**) VEGF. Data are presented as mean ± SD (*n* = 3). One-way ANOVA with Tukey’s HSD post hoc test was used; groups denoted by non-identical letters (a–g) are statistically distinct (*p* < 0.05). CP: unfermented coffee pulp; LP-CP: *Lactobacillus plantarum*-fermented coffee pulp; SC-CP: *Saccharomyces cerevisiae*-fermented coffee pulp; MIX-CP: mixed-culture fermented coffee pulp.

All coffee pulp extracts significantly upregulated Wnt/β-catenin (*CTNNB1*) and Sonic Hedgehog pathway genes (*SHH*, *SMO*, and *GLI1*) in HFDPCs relative to the untreated control (*p* < 0.05), significantly greater than minoxidil (a standard drug for hair loss) across all evaluated targets. Furthermore, MIX-CP provided the highest fold changes across all four genes (*CTNNB1*: 1.37 ± 0.03; *SHH*: 1.27 ± 0.06; *SMO*: 1.54 ± 0.05; *GLI1*: 1.70 ± 0.07), followed by LP-CP and SC-CP with comparable values, while unfermented CP exhibited the lowest expression among all treatments (Figure 10).

In addition, all fermented coffee pulp extracts significantly upregulated *VEGF* expression in HFDPCs relative to the untreated control, with MIX-CP producing the highest fold change (1.20 ± 0.07), followed by LP-CP (1.18 ± 0.09), and CP (1.12 ± 0.02). Interestingly, MIX-CP and LP-CP also significantly exceeded *VEGF* mRNA upregulation induced by minoxidil (1.06 ± 0.02), which is widely known to stimulate angiogenesis via *VEGF* upregulation (*p* < 0.05) [20].

## 3. Discussion

### 3.1. Total Phenolic Content and Antioxidant Properties of Coffee Pulp Extracts

Regarding the enzymatic mechanisms governing phenolic liberation, the increase in TPC during fermentation by lactic acid bacteria is well documented and is primarily attributed to the hydrolysis of ester bonds in phenolic acid conjugates and glycosylated polyphenols through microbial enzymes such as tannase, esterase, and glycosidases, which release bound phenolic aglycones into the free form. *L. plantarum* is particularly recognized for its robust phenolic-degrading enzymatic activity, including tannase and phenolic acid decarboxylase. The acidic environment generated by lactic acid bacteria may further facilitate acid-catalyzed hydrolysis of bound phenolics, contributing to the elevated TPC observed in LP-CP and MIX-CP treatments [21,22,23]. This progressive enhancement may be attributed to the ability of *S. cerevisiae* to promote the release and transformation of phenolic compounds from the coffee pulp matrix. During fermentation, yeast-associated hydrolytic enzymes, including esterases and glycosidases, may contribute to the cleavage of ester- or glycoside-linked phenolics, thereby increasing the amount of Folin–Ciocalteu-reactive compounds in the extract. In addition, microbial fermentation can alter the structural integrity of plant cell walls and facilitate the release of bound phenolics, while metabolic biotransformation may generate smaller phenolic derivatives or other antioxidant-related compounds. These mechanisms collectively explain the continuous increase in TPC observed in SC-CP during the 120 h fermentation period [24,25]. The comparatively lower TPC enhancement in the MIX-CP co-culture relative to LP-CP alone may indicate competitive or antagonistic metabolic interactions between *S. cerevisiae* and *L. plantarum* that partially suppress the phenolic-liberating activity of LP-CP. Co-fermentation dynamics, including pH competition and substrate partitioning, may modulate the net phenolic transformation capacity of mixed cultures [26,27,28].

Considering the overall kinetic trends observed across the fermentation timeline, the initial rise in TPC across all inoculated groups is attributable to the enzymatic activity of the microorganisms used. Both *S. cerevisiae* and *L. plantarum* secrete hydrolytic enzymes including glucosidase, amylase, cellulase, and esterase, which facilitate the structural breakdown of plant cell walls and lead to the liberation of bound phenolic compounds [29,30,31]. The peak of TPC was observed at 72–120 h, which can be explained by the competing dynamics of microbial phenolic catabolism. As fermentation progresses, microorganisms may begin to utilize released phenolic compounds as secondary carbon sources once primary sugars become limited. F. Yang et al. [32] established that lactic acid bacteria degrade polyphenols such as flavonoid glycosides, proanthocyanidins, and hydroxycinnamic acids into free aglycones and smaller phenolic molecules. While this biotransformation increases initial phenolic bioavailability, continued degradation by polyphenol-associated enzymes such as tannases, esterases, and phenolic acid decarboxylases can ultimately reduce the Folin–Ciocalteu-detectable TPC at extended fermentation durations. Similar rise-and-decline kinetics have been documented in fermented coffee pulp kombucha fermentation studies, where TPC reached a peak before declining as fermentation extended beyond an optimal window [33].

The consistently low TPC of the control group throughout the 120 h period suggests that the indigenous microflora naturally present on the coffee pulp substrate was insufficient to drive significant cell wall disruption or phenolic biotransformation at 28 °C under the conditions used. This highlights the critical role of deliberate microbial inoculation in enhancing the bioactive value of coffee pulp. The finding that LP-CP and MIX-CP consistently produced numerically higher TPC values than SC-CP alone, even where not always statistically distinct, is consistent with prior studies. Jin et al. 2019 [30] demonstrated that the co-cultivation of *L. plantarum* and *S. cerevisiae* yielded the highest TPC compared to either monoculture, attributing this to the complementary enzymatic profiles of the two organisms.

The relationship between TPC and DPPH activity provides a mechanistic basis for interpreting these findings. The close parallel between TPC and DPPH results is mechanistically expected. DPPH radical scavenging activity is primarily mediated by phenolic compounds acting as hydrogen atom donors, and the concentration of free phenolics is therefore a major determinant of DPPH activity in fermented plant substrates. This relationship has been well documented, with a strong positive correlation between TPC and DPPH activity reported across various fermented food systems [34]. In this study, the pattern supports the same relationship, as TPC increased with enzymatic phenolic release during early fermentation, DPPH activity rose correspondingly, and as TPC declined in later hours, DPPH activity followed.

The superior DPPH activity of LP-CP and MIX-CP relative to SC-CP at comparable time points reflects the well-characterized capacity of *L. plantarum* to convert glycosidically bound phenolics into their free aglycone forms. Lactobacillaceae metabolize phenolics through a suite of enzymes including reductases, decarboxylases, and glycosidases, the latter are particularly relevant as they specifically act on glycosides of plant phytochemicals including flavonoids, releasing the free aglycone forms which exhibit substantially higher radical-scavenging potency than their glycosylated precursors. This biotransformation is considered a key mechanism by which LP-CP fermentation enhances DPPH activity beyond what SC-CP alone can achieve through cell wall disruption. Additionally, *L. plantarum* produces organic acids such as lactic acid and phenyl-lactic acid during fermentation, which may further contribute to radical scavenging [35].

The decline in DPPH activity observed at extended fermentation times, especially in the SC-CP treatment, is consistent with findings reported for robusta coffee cherry fermentation, where antioxidant activity decreased with increasing fermentation duration in *S. cerevisiae*-inoculated coffee pulp, and DPPH activity was positively correlated with TPC and negatively associated with prolonged fermentation [36,37].

In addition, the upward trend in ABTS activity aligns with the general principle that microbial fermentation enhances the radical scavenging potential of plant-based matrices through the release of bound phenolics and the biotransformation of polyphenols. Similar synergistic or additive effects have been observed in studies of mango pulp and hulless barley fermented with *L. plantarum* and *S. cerevisiae*, where co-cultivation consistently outperformed monocultures [30,38].

The performance of LP-CP and MIX-CP over SC-CP in ABTS activity is again attributable to the capacity of *L. plantarum* to produce diverse antioxidant metabolites beyond phenolic release alone. *L. plantarum* produces antioxidant compounds including phenolic acids, ketones, organic acids, and exopolysaccharides (EPS) during fermentation, and also upregulates antioxidant enzymes such as superoxide dismutase (SOD) and catalase (CAT) in the fermentation matrix, collectively scavenging reactive oxygen species and contributing to radical cation deactivation [39]. The coffee pulp fermentation study by Van et al., 2023 [33] on coffee pulp kombucha similarly demonstrated that ABTS antioxidant activity tracked TPC closely during fermentation, with activity peaking and declining in patterns consistent with phenolic biotransformation dynamics, a behavior replicated in the ABTS graph of the present study. Within-group temporal variation revealed that ABTS activity did not increase monotonically but instead showed fluctuations across the 120 h. Notably, the control group exhibited its own temporal variation despite low absolute values, suggesting that even natural substrate chemistry undergoes minor changes over the fermentation duration at 28 °C, possibly due to limited activity of indigenous microflora or spontaneous oxidative processes. The inoculated groups showed more pronounced and earlier temporal shifts, reflecting the active metabolic contribution of the starter cultures. The fact that ABTS activity remained elevated in LP-CP and MIX-CP groups at 96–120 h, rather than declining sharply as observed in DPPH, may reflect the broader reactivity profile of the ABTS assay capturing sustained production of diverse radical-scavenging metabolites even as the most reactive hydrogen-donating phenolics begin to be consumed.

Regarding the electron-transfer capacity observed in the FRAP assay, this observed convergence in FRAP values likely stems from the fundamental chemical principles of the FRAP assay compared to DPPH. While the DPPH assay involves a combination of hydrogen atom transfer and single electron transfer mechanisms, making it highly sensitive to hydrogen-donating phenolic compounds, FRAP operates exclusively through a SET mechanism [36,37]. Consequently, the FRAP assay captures a more diverse spectrum of electron-donating species beyond just phenolics. During fermentation, both *S. cerevisiae* and *L. plantarum* produce an abundance of metabolic byproducts, including organic acids (such as lactic acid) and small-molecular-weight metabolites, which may not be detected by the Folin–Ciocalteu reagent used in TPC measurements. The reductive capacity common to these shared fermentation metabolites is sufficient to elevate FRAP values uniformly across all inoculated cultures. In contrast, the DPPH assay’s specificity for hydrogen-donating capacity makes it more sensitive to the qualitative differences in phenolic biotransformation between lactic acid bacteria and yeast.

### 3.2. Global Metabolomic Profiling and Multivariate Statistical Analysis of Fermented Coffee Pulp

The overall metabolomic alterations across treatments provide insight into the distinct effects of the different fermentation strategies. Under the stringent statistical criteria, LP-CP produced the largest number of significantly altered metabolite features, whereas MIX-CP was characterized by a predominantly upward direction among its significantly altered features (84.10% increased), despite comprising a smaller overall number of altered features. This pattern is corroborated by the multivariate analysis: the clear separation of control from fermented groups along PC1 suggests that fermentation was the major driver of metabolomic variation, and the pronounced separation of LP-CP along PC1 is consistent with its greater number of significantly altered metabolite features, while SC-CP and MIX-CP occupied distinct positions along PC2 and PC3, indicating that each fermentation strategy generated a characteristic pattern of metabolic remodeling rather than a uniformly greater overall shift in MIX-CP.

### 3.3. Polyphenol Profile of Fermented Coffee Pulp

Regarding the polyphenol composition observed in the results, the higher concentration of specific compounds in LP-CP is consistent with the well-documented ability of Lactobacillus plantarum to hydrolyze glycosidically bound polyphenols through tannase and β-glucosidase activity, thereby liberating free flavan-3-ol aglycones such as catechin and epicatechin from the coffee pulp matrix [6,7].

On the other hand, the presence of specific key active components in MIX-CP plays a crucial role in its bioactivity. Rosmarinic acid and quercetin are well recognized for their potent antioxidant and anti-inflammatory activities and have been reported to suppress 5α-reductase activity while promoting HFDPC proliferation, two mechanisms closely associated with hair growth [40]. This finding may explain the superior biological activity of MIX-CP across all in vitro assays, despite LP-CP exhibiting a more abundant polyphenol profile in the HPLC analysis. The co-fermentation environment of MIX-CP, combining *L. plantarum* and *S. cerevisiae*, likely promotes a broader and more balanced spectrum of polyphenol derivatives together with other non-phenolic antioxidant metabolites, such as organic acids and exopolysaccharides, that may exert complementary effects rather than relying on the accumulation of any single dominant compound [30]. These results suggest that the hair-growth-promoting potential of fermented coffee pulp extracts may be governed less by the absolute abundance of major polyphenols and more by the diversity and complementary contributions of bioactive metabolites generated through mixed-culture fermentation.

### 3.4. Cytotoxic Assessment and Proliferative Response of Cells Treated with Fermented Coffee Pulp Extracts

To explain the mechanisms underlying the proliferative outcomes and metabolomic patterns observed in the results, the enhanced HFDPC proliferative activity of MIX-CP may reflect qualitative compositional differences arising from the complementary metabolic activities of *S. cerevisiae* and *L. plantarum*. Whereas *L. plantarum* drives the hydrolysis of ester-linked and glycosylated phenolic conjugates through tannase, esterase, and glycosidase activity—substantially increasing the pool of free bioavailable phenolic aglycones as reflected in the elevated highest TPC and antioxidant activities values of LP-CP described previously—*S. cerevisiae* contributes through cell wall-degrading enzymes and the production of diverse metabolic byproducts, including organic acids and small-molecular-weight reductants that broaden the scope of antioxidant capacity uniformly across all fermented treatments [21,22,23,41]. The combined metabolic activities in MIX-CP may therefore generate a qualitatively distinct bioactive milieu compared with either monoculture, which may contribute to the observed proliferative response of HFDPCs at low concentrations without imposing a cytotoxic burden at the selected experimental dose.

Furthermore, considering the biological significance of these cellular responses, HFDPCs are pivotal regulators of hair follicle morphogenesis, coordinating the induction, organogenesis, and cytodifferentiation stages through reciprocal paracrine signaling with epithelial, keratinocyte, and fibroblast cell populations. The pro-proliferative capacity of MIX-CP on HFDPCs at concentrations well below the cytotoxic threshold suggests a favorable safety-to-activity window and highlights its potential to support hair follicle function and promote hair growth [42].

### 3.5. Effect of HFDPC-Conditioned Medium from Fermented Coffee Pulp Extracts on Human Fibroblast Proliferation

To elucidate the biological mechanisms underlying the functional outcomes observed in the results, a plausible contributing mechanism, based on established HFDPC biology, is the secretion of growth factors and morphogenic ligands by MIX-CP-stimulated HFDPCs. In the hair follicle microenvironment, perifollicular dermal fibroblasts play a critical role in synthesizing extracellular matrix components and providing structural support necessary for hair follicle development and cycling [43,44]. VEGF is a well-established paracrine mediator secreted by HFDPCs that promotes angiogenesis and supports this fibroblast activation in the perifollicular dermis, contributing to the maintenance of a vascularized and metabolically active hair follicle environment conducive to fibroblast proliferation. Furthermore, Wnt ligands secreted by HFDPCs constitute a canonical signal that activates β-catenin-dependent transcriptional programs in adjacent fibroblasts, promoting their proliferation and supporting dermal remodeling associated with hair follicle cycling [45]. Consequently, the enhanced proliferation and metabolic activity observed in neighboring fibroblasts exposed to MIX-CP-primed HFDPC-conditioned medium may reflect a strengthened perifollicular dermal niche, potentially supporting improved nutrient delivery via enhanced vascularization. The greater ability of MIX-CP to enhance fibroblast proliferation through conditioned medium relative to monoculture extracts suggests that the compositionally distinct bioactive milieu generated by co-fermentation may more effectively prime HFDPCs toward upregulation of these candidates signaling mediators, although this remains to be directly confirmed.

However, these cellular outcomes should be interpreted in light of certain methodological considerations. The conditioned medium collection protocol does not completely remove residual low-molecular-weight extract compounds. Therefore, the observed effects may reflect both HFDPC-derived paracrine signaling and direct effects of residual extract components. Future studies should include an extract-only control, in which the extract is processed under the same conditions without HFDPCs, to distinguish the contribution of residual extract components from HFDPC-mediated paracrine effects.

In terms of the comparative performance across different fermentation strategies, the comparatively lower conditioned-medium-induced proliferative activity of SC-CP relative to both LP-CP and MIX-CP aligns with the established mechanism by which *L. plantarum* enhances the free phenolic aglycone pool through tannase, esterase, and glycosidase activity—generating bioavailable phenolic species with documented capacity to modulate cellular signaling in dermal fibroblast cells [46,47]. In contrast, *S. cerevisiae* monoculture fermentation, while contributing to overall metabolite diversity through cell wall-degrading enzymes and organic acid production, does not replicate the phenolic biotransformation depth achieved by lactic acid bacteria. The convergence of both enzymatic profiles in MIX-CP thus generates a conditioned medium with the highest fibroblast-proliferative activity among the conditions tested, suggesting mixed-culture fermentation as the optimal strategy for maximizing the indirect pro-proliferative activity of coffee pulp extracts on dermal fibroblasts.

### 3.6. Protective Effects of Fermented Coffee Pulp Extracts Against Tolbutamide-Induced Reduction in HFDPC Viability

The present findings demonstrate that fermented coffee pulp extracts, particularly MIX-CP, attenuate the reduction in HFDPC viability following tolbutamide exposure. However, because the assay measured cellular protein content using the SRB method rather than channel current, membrane potential, or K_ATP_-channel activity, the mechanism underlying this protective response cannot be determined from the present data. The observed effect may involve cytoprotective mechanisms unrelated to direct K_ATP_-channel modulation [48].

MIX-CP produced a greater recovery of HFDPC viability than the other extracts under the tested conditions. This response was not directly correlated with TPC or antioxidant capacity, suggesting that qualitative differences in metabolite composition may contribute to the observed cytoprotective effect. However, the specific metabolites and molecular mechanisms responsible remain to be identified. Electrophysiological or membrane potential measurements would be required to determine whether MIX-CP directly affects K_ATP_-channel function.

### 3.7. Anti-Inflammatory Properties of Fermented Coffee Pulp Extracts in Cell-Based Models

The mechanistic implications of these anti-inflammatory trends should be considered in the context of the assay-specific response patterns. The ranking observed in this assay—where LP-CP leads and MIX-CP closely follows without significant difference—differs from the patterns observed in the K_ATP_ and ROS assays but aligns closely with the antioxidant capacity and TPC profiles LP-CP’s superior activity is consistent with its enhanced polyphenol content (Section 2.4), in line with established evidence that polyphenols suppress LPS-induced NO overproduction through inhibition of iNOS transcription and downstream inflammatory signaling. The near-equivalent performance of MIX-CP suggests that the *L. plantarum*-derived phenolic fraction remains the primary driver of anti-inflammatory activity in the co-fermentation system, while yeast-derived metabolites contribute in a complementary but non-additive manner [21,22,23,49].

In the broader context of hair follicle pathophysiology, the anti-inflammatory activity of fermented coffee pulp extracts in both HFDPCs and RAW 264.7 macrophages, two cell types relevant to the follicular and perifollicular inflammatory microenvironments in alopecia, suggests that *L. plantarum*-mediated phenolic biotransformation is the primary driver of anti-inflammatory bioactivity in this extract series. Co-fermentation with *S. cerevisiae* in MIX-CP appears to preserve this activity while broadening the overall metabolite spectrum of the extract. By effectively suppressing NO-mediated inflammatory cascades in both follicular and perifollicular cells, these fermented extracts demonstrate significant potential to mitigate the microinflammation-driven follicle miniaturization and premature anagen-to-catagen transition that characterize pathological hair loss, thereby offering a strategic therapeutic approach for preserving hair follicle homeostasis and promoting sustained hair growth [15].

### 3.8. Suppression of Intracellular Reactive Oxygen Species by Fermented Coffee Pulp Extracts

The relationship between chemical antioxidant assays and intracellular activity provides an important context for interpreting the ROS findings. The superior intracellular ROS-suppressing activity of MIX-CP relative to LP-CP in this assay is particularly noteworthy, as it represents a clear dissociation from the antioxidant profiling data in which LP-CP consistently exhibited the highest TPC and antioxidant activity values. This divergence indicates that cell-free radical scavenging capacity does not translate directly to intracellular ROS suppression in HFDPCs and that the biological determinants of cellular antioxidant efficacy extend beyond the abundance of phenolic constituents generated during bacterial fermentation. In this respect, the ROS data parallel the findings from the K_ATP_ channel assay, where a comparable dissociation between TPC and functional cellular activity was observed, reinforcing the interpretation that co-fermentation generates a qualitatively distinct bioactive composition—one that neither yeast nor bacterial monoculture fermentation can replicate—whose cellular efficacy cannot be predicted from bulk antioxidant measurements alone [50].

The metabolomic data provide additional context for interpreting the intracellular ROS findings. MIX-CP exhibited fewer significantly altered features than LP-CP, despite showing the greater ROS-lowering effect under the conditions tested. This comparison indicates that the magnitude of intracellular ROS suppression was not simply proportional to the total number of significantly altered metabolite features. The difference may reflect the biological properties, cellular uptake, stability, or intracellular activity of specific metabolites rather than the overall number of metabolomic changes. However, the present untargeted analysis does not identify which metabolites contribute to this response, and targeted metabolite identification together with functional validation will be required to clarify the underlying mechanism. The observed reduction in intracellular ROS supports further investigation of MIX-CP in cell-based oxidative-stress models, but does not by itself establish protection against hair-follicle dysfunction [3].

### 3.9. Suppression of Lipid Peroxidation in H_2_O_2_-Challenged HFDPCs by Fermented Coffee Pulp Extracts

The biochemical basis of these protective outcomes is further supported by the superior lipid peroxidation-suppressing activity of mixed-culture fermented MIX-CP relative to bacterial monoculture fermented LP-CP in this assay. This finding is consistent with the pattern observed across multiple cell-based endpoints in this study and again represents a dissociation from the bulk antioxidant profiling data in which LP-CP exhibited higher TPC and all antioxidant activity values. This recurring divergence is consistent with the interpretation that co-fermentation generates a qualitatively distinct bioactive composition—encompassing not only phenolic aglycones liberated by *L. plantarum* enzymatic activity but also *S. cerevisiae*-derived metabolites, including organic acids and low-molecular-weight reductants—that may collectively contribute to membrane-protective effects in HFDPCs through complementary molecular interactions, a pattern not observed with bacterial monoculture fermentation alone in this study [50]. These findings are further consistent with the intracellular ROS suppression data reported in Section 2.8, where MIX-CP produced a greater reduction in intracellular ROS than LP-CP despite the latter’s higher cell-free antioxidant capacity, collectively indicating that the co-fermentation-derived bioactive milieu attenuates oxidative stress in HFDPCs through convergent mechanisms targeting both ROS generation and downstream lipid peroxidation cascades.

In the broader context of hair follicle maintenance, the capacity of MIX-CP to reduce MDA accumulation to levels approaching the pharmacological antioxidant reference in H_2_O_2_-challenged HFDPCs underscores the translational relevance of mixed-culture fermentation in generating coffee pulp extracts with meaningful cellular antioxidant protection. By attenuating lipid peroxidation-driven membrane damage in HFDPCs, co-fermented coffee pulp extracts may help support the structural and functional integrity of dermal papilla cells under oxidative microenvironmental conditions characteristic of inflammatory and AGA, thereby supporting the maintenance of HFDPC paracrine signaling capacity and the preservation of anagen-phase hair follicle function [51]. These in vitro findings collectively indicate that MIX-CP exhibited consistently favorable activity across the oxidative stress-related endpoints assessed in this study, warranting further mechanistic investigation into the specific intracellular pathways engaged by co-fermentation-derived bioactives in HFDPC cytoprotection.

### 3.10. Transcriptional Modulation of Hair Loss-Related Genes in the Androgen and TGF-β Signalling Pathways by Fermented Coffee Pulp Extracts

The mechanistic significance of the *SRD5A* down-regulation observed in the quantitative analysis can be considered in the context of the greater *SRD5A* mRNA suppression observed with fermented coffee pulp extracts relative to established pharmacological standards. This finding is consistent with the enriched free phenolic content generated through microbial biotransformation, as polyphenolic compounds have been reported to inhibit SRD5A enzyme activity through competitive binding interactions at the enzyme active site. These findings support the transcriptional regulation of *SRD5A1* and *SRD5A2*, while further functional studies are warranted to assess the corresponding enzymatic effects. The convergence of complementary bioactive classes in MIX-CP—encompassing phenolic aglycones liberated by *L. plantarum* enzymatic activity alongside *S. cerevisiae*-derived metabolites—may contribute to the broader *SRD5A1* and *SRD5A2* mRNA suppression through multiple complementary molecular interactions, potentially conferring broader isoenzyme-transcript coverage than the monoculture-fermented counterparts [46].

The downstream anti-apoptotic implications of TGFB1 suppression are also relevant to the follicular microenvironment. The capacity of MIX-CP and LP-CP to suppress *TGFB1* to a greater extent, at the mRNA level, than that observed with the standard treatments used in this study—agents that act exclusively at the upstream SRD5A enzymatic step—suggests that fermented coffee pulp extracts may additionally modulate *TGFB1* transcriptional regulation through mechanisms independent of DHT-mediated induction, potentially involving antioxidant- or anti-inflammatory-related activity on HFDPC signaling networks, consistent with the superior NO-suppressing and ROS-attenuating activities documented in Section 3.6 and Section 3.7. The suppression of *TGFB1* mRNA expression may have functional relevance to hair follicle biology, as reduced TGF-β1 signaling has been reported to attenuate the apoptotic cascade in follicular epithelial cells, thereby extending the anagen phase and counteracting the premature follicular regression characteristic of AGA pathogenesis.

Taken together, these findings indicate that MIX-CP modulates the transcriptional expression of both upstream (*SRD5A1* and *SRD5A2*) and downstream (*TGFB1*) mediators relevant to androgenetic hair loss at the mRNA level, though corresponding enzyme activity and protein-level confirmation remain to be established.

### 3.11. Transcriptional Modulation of Hair Growth-Related Genes via Wnt/β-Catenin, Sonic Hedgehog, and Angiogenic Signaling Pathways by Fermented Coffee Pulp Extracts

Regarding the transcriptional responses observed in the morphogenic pathways, the broadly comparable upregulation of Wnt/β-catenin and Sonic Hedgehog pathway genes across all fermented groups—with MIX-CP consistently exhibiting the highest expression levels—indicates that these hair growth-promoting transcriptional programmes are responsive to the general increase in bioavailable species and fermentation-derived metabolites shared across all inoculated treatments, rather than being selectively driven by the enzymatic output of any single microbial strain. Crucially, the upregulation of these core pathway transcripts is also relevant to the regulation of downstream angiogenic pathways, as canonical Wnt/β-catenin signaling has been reported to act as a transcriptional driver for the expression of vascular effectors within the dermal papilla microenvironment [52].

Consequently, VEGF is an indispensable angiogenic mediator during the hair follicle anagen phase, functioning to establish and sustain the perifollicular microvasculature required for adequate oxygen and nutrient delivery to the metabolically active dermal papilla [53]. *VEGF* upregulation in HFDPCs has been reported to increase follicular diameter, prolong anagen duration, and accelerate hair shaft growth by enhancing the vascular niche that supports HFDPC signaling and follicular keratinocyte proliferation [53]. Based on this established biology, VEGF induction in HFDPCs may plausibly link the transcriptional activation of hair growth pathways observed here with the fibroblast proliferation response described in Section 2.5, although this connection was not directly tested in the present study.

The increase in *VEGF* mRNA is consistent with a possible contribution of HFDPC-derived signaling to the conditioned-medium response described in Section 2.5, where MIX-CP-conditioned medium produced the greatest stimulation of fibroblast proliferation. However, it does not establish VEGF protein secretion or confirm that the fibroblast response was mediated by VEGF or other paracrine factors.

Taken together, the gene expression data across all assessed pathways indicate a coordinated, multi-target transcriptional response potentially relevant to androgenetic hair loss. MIX-CP simultaneously suppressed mRNA expression of the upstream androgen biosynthesis genes *SRD5A1* and *SRD5A2*, attenuated the downstream pro-regression mediator TGF-β1, and upregulated hair follicle growth-related transcriptional programmes encompassing Wnt/β-catenin (*CTNNB1*), Sonic Hedgehog (*SHH*, *SMO*, *GLI1*), and angiogenic (*VEGF*) pathways, with changes numerically comparable to or greater than those seen with the pharmacological reference standards at the mRNA level. This multi-pathway transcriptional profile was most pronounced in MIX-CP relative to either bacterial or yeast monoculture fermentation alone, which may reflect the greater metabolite diversity generated under co-fermentation conditions. However, these findings are based on mRNA-level changes only; corresponding protein, enzyme activity, and functional evidence—together with validation in more complex biological models and, ultimately, clinical evaluation—would be required before mixed-culture fermented coffee pulp could be considered a candidate for the prevention or management of AGA.

## 4. Materials and Methods

### 4.1. Chemicals and Reagents

Analytical-grade reagents were used throughout all investigations. Folin–Ciocalteu reagent, aluminum chloride hexahydrate, hydrogen peroxide (H_2_O_2_), trichloroacetic acid (TCA), and Triton X-100 were supplied by Merck (Darmstadt, Germany). Reagents from Sigma-Aldrich (St. Louis, MO, USA) included 2,2′-azino-bis (3-ethylbenzothiazoline-6-sulfonic acid) (ABTS), 2,2-diphenyl-1-picrylhydrazyl (DPPH), sulforhodamine B (SRB), lipopolysaccharide (LPS), and dimethyl sulfoxide (DMSO). Thiobarbituric acid (TBA) was obtained from VWR Chemicals (BDH Chem. Ltd., Poole, UK). Standard compounds and positive controls—epigallocatechin gallate (EGCG), gallic acid, Trolox (6-hydroxy-2,5,7,8-tetramethylchroman-2-carboxylic acid), diclofenac sodium, and L-ascorbic acid—were also purchased from Sigma-Aldrich. The standard compounds for hair regeneration (dutasteride, finasteride, minoxidil, and purmorphamine) were purchased from Wuhan W&Z Biotech (Wuhan, China).

For the cell culture, Dulbecco’s Modified Eagle Medium (DMEM), Roswell Park Memorial Institute 1640 Medium (RPMI-1640), fetal bovine serum (FBS), and penicillin-streptomycin solution (100×) were purchased from Gibco Life Technologies (Thermo Fisher Scientific, Waltham, MA, USA). The Fibroblast Cell Growth Medium was purchased from Promo Applied Biological Materials Inc. (Richmond, BC, Canada). The Griess reagent colorimetric assay kit was purchased from Invitrogen (Thermo Fisher Scientific, Inc., Eugene, OR, USA). Human hair follicle dermal papilla cells (HFDPCs; catalog no. 2502) were acquired from iCell Bioscience Inc. (Shanghai, China). Murine macrophage cells (RAW 264.7; ATCC TIB-71), human prostate cancer cells (DU-145; ATCC HTB-81), and human dermal fibroblast cells (hTERT; ATCC CRL-4066) were obtained from the American Type Culture Collection (Rockville, MD, USA).

Software used for data analysis included LabSolutions version 1.86 (Shimadzu, Kyoto, Japan), ImageJ version 1.53t (NIH, Bethesda, MD, USA), and Image Lab™ version 5.1 (Bio-Rad Laboratories, Hercules, CA, USA).

### 4.2. Extract Preparation

#### 4.2.1. Coffee Pulp: Source and Powder Preparation

Coffee pulp from Arabica coffee (*Coffea arabica* L.) was kindly provided by the Nong Hoi Highland Research Station, Faculty of Agriculture, Chiang Mai University, Chiang Mai, Thailand (18.929670491361765, 98.8161849920434).

The coffee pulp was dried in a hot-air oven at 50 °C for 12 h until a constant weight was achieved, then ground using a laboratory mill and passed through a 425 µm stainless steel mesh sieve to obtain a uniformly fine powder. The powder was stored in sealed polyethylene bags at room temperature in a dark, dry environment until use.

#### 4.2.2. Microbial Strains: Source and Inoculum Preparation

Two microbial strains (*Saccharomyces cerevisiae* TISTR 5169 and *Lactobacillus plantarum* TISTR 2072) were purchased from the Thailand Institute of Scientific and Technological Research (TISTR), Bangkok, Thailand.

To prepare the microbial cultures, each microbial strain was cultivated separately in an appropriate liquid growth medium under optimized conditions. *S. cerevisiae* TISTR 5169 was propagated in Yeast Extract Peptone Dextrose (YPD) broth (10 g/L yeast extract, 20 g/L peptone, 20 g/L dextrose) and incubated at 30 °C for 24 h. *L. plantarum* TISTR 2072 was cultivated in De Man, Rogosa and Sharpe (MRS) broth (Himedia, Mumbai, India) at 37 °C for 24 h under anaerobic conditions.

Following incubation, microbial cells were harvested by centrifugation at 5000× *g* for 10 min at 4 °C. The resulting cell pellets were washed twice with sterile 0.85% (*w*/*v*) NaCl to remove residual growth medium. The washed cells were resuspended in sterile distilled water and subjected to freeze-drying using a vacuum freeze-dryer. The resulting freeze-dried microbial powders were stored at −20 °C until further use.

For single-culture fermentation, each freeze-dried microbial powder was reconstituted individually in sterile distilled water at a concentration of 0.1% (*w*/*v*) to produce single-organism inocula for *S. cerevisiae* and *L. plantarum*, respectively. For mixed-culture fermentation, the two freeze-dried powders were dissolved together in sterile distilled water at a combined concentration of 0.1% (*w*/*v*) and mixed at an equal ratio of 1:1 (*S. cerevisiae*: *L. plantarum*) by weight. All inocula were freshly prepared immediately prior to use and maintained on ice until inoculation of the fermentation substrate.

#### 4.2.3. Fermentation

The experiment was conducted using Completely Randomized Design (CRD) with four treatment groups and three replicates per treatment (*n* = 3). The experimental treatments were designed as shown below(Table 4).

**Table 4:** Experimental treatment groups for coffee pulp fermentation. Inoculation rates are expressed as the volume of inoculum solution added per unit weight of substrate (%, *v*/*w*).

Treatment	Inoculation Rate (%, *v*/*w*)	
	*S. cerevisiae*	*L. plantarum*
CP	0	0
LP-CP	0	1
SC-CP	1	0
MIX-CP	0.5	0.5

Abbreviations: CP, unfermented coffee pulp; LP-CP, *Lactobacillus plantarum*-fermented coffee pulp; SC-CP, *Saccharomyces cerevisiae*-fermented coffee pulp; MIX-CP, mixed-culture fermented coffee pulp.

Fifty grams of coffee pulp powder was weighed into each sterile glass fermentation jar, and 40 mL of sterile distilled water was added, corresponding to a coffee pulp powder-to-water ratio of 50 g:40 mL (1:0.8, *w*/*v*). The mixture was thoroughly mixed to obtain a homogeneous slurry before inoculation. For the single-culture treatments, the respective microbial inoculum was added at 1% (*v*/*w*) relative to the substrate weight. For the mixed-culture treatment (MIX-CP), *S. cerevisiae* and *L. plantarum* were each added at 0.5% (*v*/*w*), resulting in a total inoculation rate of 1% (*v*/*w*). The inocula were mixed thoroughly with the substrate to ensure even distribution. The jars were sealed with gas-permeable caps to allow CO_2_ release while preventing contamination. Fermentation was carried out at 28 °C for 120 h. Subsequently, all fermented materials were transferred to stainless steel trays and dried in a hot-air oven at 50 °C until a constant weight was achieved. The dried fermented coffee pulp was milled and passed through a 425 µm stainless steel mesh sieve to yield a fine, uniform powder. All powders, including the unfermented control, were stored at −20 °C in sealed containers prior to extraction.

#### 4.2.4. Ultrasonic-Assisted Extraction

Fermented coffee pulp powder from each treatment group was extracted by ultrasonic-assisted extraction. Each powder was suspended in 95% (*v*/*v*) ethanol at a solid-to-solvent ratio of 1:5 (*w*/*v*) and mixed thoroughly to ensure complete suspension. Extraction was performed in an ultrasonic bath at 40 kHz for 20 min and repeated twice; the bath was maintained under cooled conditions throughout to minimize thermal degradation of thermolabile bioactive compounds. The extracts were filtered through a Whatman filter paper to remove particulate matter. The filtrates were then concentrated under reduced pressure using a rotary evaporator (Rotavapor^®^ R-300, Büchi, Flawil, Switzerland) at 200 mbar and 120 rpm with a 30 °C water bath until a final volume of 15 mL was reached. The resulting concentrated extracts were stored at −20 °C, protected from light, until analysis.

### 4.3. Phytochemical Analysis

#### 4.3.1. Total Phenolic Content

Total phenolic content was determined using a modified Folin–Ciocalteu colorimetric assay [42]. Firstly, the coffee pulp extract was combined with 10% (*v*/*v*) Folin–Ciocalteu reagent in a 96-well microplate. After 5 min, 6% (*w*/*v*) saturated sodium bicarbonate solution was added to neutralize the reaction mixture. Afterward, the plate was incubated in darkness at room temperature for 2 h. The absorbance was measured at 765 nm using a UV-Vis spectrophotometer (EZ Read 400 Flexi, Biochrom, Cambridge, UK). A calibration curve was determined using gallic acid standards (GAE) ranging from 10 to 200 µg/mL. Results were expressed as milligrams of gallic acid equivalents per gram of coffee pulp extract (mg GAE/g coffee pulp extract).

#### 4.3.2. Antioxidant Activities

• ABTS Radical Scavenging Activity

The ABTS assay was used to determine the free radical scavenging capabilities of the coffee pulp extracts [42]. The ABTS radical cation (ABTS•+) (stock solution) was prepared by combining equal volumes of 7.0 mM ABTS solution with 2.45 mM potassium persulfate solution. The stock solution was stored in the dark at room temperature (25 ± 2 °C) for 12–16 h, and freshly prepared stock solution was used. The working solution was prepared by diluting the stock solution with 95% (*v*/*v*) ethanol to achieve an absorbance of 0.70 ± 0.02 at 734 nm. For the assay, the samples were combined with working solution in a 96-well microplate and incubated in the dark for 30 min at room temperature (25 ± 2 °C). The absorbance was measured at 734 nm using a UV-Vis spectrophotometer. Gallic acid was used as the standard, and 95% (*v*/*v*) ethanol was used as the blank control. ABTS radical-scavenging capacity was calculated from the gallic acid calibration curve and expressed as milligrams of gallic acid equivalents per gram of extract (mg GAE/g). The use of gallic acid as the calibration standard represented a modification of the originally described ABTS method.

• DPPH Radical Scavenging Activity

The DPPH assay was used to determine the free radical scavenging capabilities of the coffee pulp extracts [42]. A solution of 0.1 nM DPPH was prepared in 95% (*v*/*v*) ethanol. Afterward, the samples were combined with 0.1 mM DPPH solution in a 96-well microplate and incubated in the dark at room temperature (25 ± 2 °C) for 30 min. The absorbance was measured at 515 nm using a UV-Vis spectrophotometer. Gallic acid was used as the standard, and 95% (*v*/*v*) ethanol was used as the blank control. DPPH radical-scavenging capacity was calculated from the gallic acid calibration curve and expressed as milligrams of gallic acid equivalents per gram of extract (mg GAE/g). The use of gallic acid as the calibration standard represented a modification of the originally described DPPH method.

• Ferric Reducing Antioxidant Power (FRAP) Assay

The ferric reducing antioxidant power (FRAP) of each extract was determined according to Benzie and Strain [42]. The FRAP reagent was freshly prepared by mixing 300 mM acetate buffer (pH 3.6), 10 mM TPTZ dissolved in 40 mM HCl, and 20 mM FeCl_3_·6H_2_O in deionized water at a ratio of 10:1:1 (*v*/*v*/*v*) and warmed to 37 °C prior to use. For the assay, 20 µL of appropriately diluted sample was added to a 96-well microplate, followed by 180 µL of FRAP reagent. The mixture was incubated at room temperature for 5 min in the dark, and absorbance was measured at 595 nm. FRAP values were calculated from an Fe (II) calibration curve and expressed as μmol Fe^2+^ equivalents per gram of extract (μmol Fe^2+^/g extract). All analyses were performed in triplicate.

### 4.4. Untargeted Metabolomic Profiling

#### 4.4.1. Metabolite Extraction and Sample Preparation

Metabolites were extracted from samples following a published protocol for plant metabolomics with minor modifications [54,55]. Briefly, samples were centrifuged at 14,000× *g* for 20 min at 16 °C to pellet insoluble material. The clarified supernatant was then passed through an ultrafiltration membrane with a 3 kDa molecular-weight cut-off to remove proteins and other macromolecules, thereby enriching for small-molecule metabolites (<3 kDa). The filtrate was subjected to solid-phase extraction (SPE) on C_18_ cartridges mounted on a vacuum manifold. SPE cartridges were pre-conditioned with 30 mL of acetonitrile, then equilibrated with 60 mL of deionized water. Each sample filtrate was loaded onto a cartridge, which was subsequently washed with water and then eluted with 99% acetonitrile in water. The eluted metabolites were concentrated by rotary evaporation under vacuum until dry. Finally, the dried extract was reconstituted in 1 mL of methanol and diluted 1:10 with deionized water to a final volume of 10 mL.

#### 4.4.2. UHPLC–MS/MS Conditions for Untargeted Metabolomics

Untargeted metabolomic profiling was performed using an UltiMate 3000 UHPLC system (Thermo Fisher Scientific) coupled to a Thermo Q Exactive-X Orbitrap mass spectrometer. Chromatographic separation was achieved on a Hypersil GOLD™ C18 column (2.1 mm × 100 mm, particle size 1.9 µm) maintained at 60 °C. For each analysis, 3 µL of sample was injected, and the UHPLC flow rate was 0.35 mL/min. The auto-sample was kept at 8 °C to prevent sample degradation. The MS was operated in both positive and negative electrospray ionization (ESI) modes, so two corresponding solvent systems were used. In positive mode, mobile phase A was 10:90 methanol/water with 0.1% formic acid and 10 mM ammonium formate, while mobile phase B was 90:5 acetonitrile/water with 0.1% formic acid and 10 mM ammonium formate. In negative mode, mobile phase A was 10:80 (*v*/*v*) methanol/water with 0.1% acetic acid, and mobile phase B was 90:10 (*v*/*v*) acetonitrile/water with 0.1% acetic acid. The gradient program was as follows: initially 95% A and 5% B, held for 2 min; then a linear ramp to 55% B over the next 6 min. The column was then flushed with 99% B for 5 min to wash off late-eluting compounds and finally returned to the initial 95% A/5% B conditions for re-equilibration. Total run time was 20 min per sample. To prevent carryover between samples, a blank injection (methanol) was run between each biological sample run.

The mass spectrometer’s ESI source was set to 3.5 kV spray voltage in positive mode and 3.2 kV in negative mode. Sheath gas and auxiliary gas were set at 50 and 10 (arbitrary units), respectively. The capillary temperature was 350 °C. The Orbitrap analyzer alternated between full MS scans and data-dependent MS/MS (dd-MS2) scans. Full MS spectra were acquired at 180,000 resolving power (at *m*/*z* 200) across a mass range of *m*/*z* 85–850. The automatic gain control (AGC) target for full MS was set to achieve optimal ion accumulation (with the instrument’s default setting ensuring <1 ppm mass accuracy by using an internal calibrant). For MS/MS acquisition, the twenty most intense precursor ions from each full scan were isolated (within a ±1.4 Da window) and fragmented by higher-energy collisional dissociation (HCD). The HCD collision energy was stepped or optimized per ion based on intensity. Dynamic exclusion was enabled to avoid repeatedly fragmenting the same ion within a short time window. The MS/MS scans were collected at 30,000 resolving power. All instrument control and data acquisition were performed using Thermo Xcalibur 3.1 software.

#### 4.4.3. Data Processing and Metabolite Identification

Raw LC–MS/MS data files were imported into Compound Discoverer 3.1 (Thermo Fisher Scientific) for data processing using an untargeted metabolomics workflow. Features (ion signals) were detected and aligned across samples with the following parameters: a retention time alignment tolerance of 0.4 min and mass tolerance of 5 ppm for peak matching. A minimum signal-to-noise ratio of 1.5 was required for feature detection, and an intensity variation tolerance of 30% was applied when aligning peaks. To reduce technical variation, a QC-based signal correction (cubic spline regression model) was applied using pooled quality-control runs. Features with peak intensity below 5 × 10^5^ (arbitrary units) were considered noise and excluded. For each detected feature, the software predicted possible molecular formulas based on accurate mass (with a tolerance of 5 ppm) and isotopic pattern. These candidate formulas were then compared against multiple online databases to aid identification. MS/MS fragmentation spectra were searched in the mzCloud library (https://www.mzcloud.org, accessed 18 March 2026) for best-fit compound matches. In addition, formulae and accurate masses were queried in ChemSpider (http://www.chemspider.com, accessed 18 March 2026), which aggregates entries from sources such as ChEBI and ChemBank for endogenous plant metabolites [56]. To capture secondary metabolites, we also referenced The Natural Products Atlas 3.0 database (accessed 18 March 2026) for any hits corresponding to natural products [57]. Each feature was thus annotated with putative identifications, and the top-ranking annotation (highest spectral match score, with mass error <2 ppm and plausible chemistry) was retained as the most confident identification. All putative identifications were manually reviewed to ensure consistency of retention time and fragmentation pattern with the proposed structure.

### 4.5. Polyphenol Profile Analysis

Polyphenol composition was determined based on a previously reported protocol [42], with minor modifications. Each extract was first diluted in 50% (*v*/*v*) ethanol and clarified by passing through a 0.45 μm syringe filter prior to injection. Analysis was carried out on a Shimadzu high-performance liquid chromatography (HPLC) system fitted with a photodiode array detector (CTO-20AC, Shimadzu, Kyoto, Japan), monitored at a detection wavelength of 280 nm. Chromatographic separation was performed in reverse-phase mode using a Restek Ultra C18 column (250 × 4.6 mm, 5 μm; Restek, Bellefonte, PA, USA), maintained at a column temperature of 40 °C. The mobile phase consisted of two solvents: solvent A (95% water, 5% formic acid) and solvent B (85% acetonitrile, 10% water, 5% formic acid), delivered at a flow rate of 1 mL/min with an injection volume of 10 μL. Separation was achieved using the following gradient elution program: 20% B maintained from 0 to 4 min, increased to 75% B from 4 to 12 min, held at 75% B from 12 to 14 min, then decreased stepwise to 30% B (14–17 min) and further to 5% B (17–18 min). The identification of individual polyphenols was based on the comparison of their retention times and UV spectra with those of authentic standards.

### 4.6. In Vitro Cell Viability and Proliferation Assay

Human hair follicle dermal papilla cells (HFDPCs) were maintained in Fibroblast Cell Growth Medium. Murine macrophages (RAW 264.7) and human fibroblasts (hTERT) were cultured in DMEM, while human prostate cancer (DU-145) cells were grown in RPMI-1640. All media were supplemented with 10% (*v*/*v*) FBS and 1% (*v*/*v*) penicillin–streptomycin (100×). Cells were incubated at 37 °C in a humidified atmosphere containing 5% CO_2_. Cell viability and proliferation were assessed using the SRB colorimetric assay [58]. Briefly, cells were seeded into 96-well plates at a density of 1 × 10^5^ cells/mL and allowed to adhere for 24 h. After attachment, cells were treated with the unfermented coffee pulp extract (CP), 120 h fermented coffee pulp extracts (LP-CP, SC-CP, and MIX-CP), and the standard compounds (diclofenac sodium, L-ascorbic acid, dutasteride, finasteride, minoxidil, and purmorphamine) at concentrations ranging from 0.004 to 2.000 mg/mL for an additional 24 h. Following treatment, the culture medium was removed, and cells were fixed with ice-cold 50% (*w*/*v*) TCA for 1 h at 4 °C. Afterward, fixed cells were stained with 0.04% (*w*/*v*) SRB solution for 30 min at room temperature (25 ± 2 °C). Excess dye was removed by washing with 1% (*v*/*v*) acetic acid, and the protein-bound dye was solubilized in 10 mM Tris base solution (pH 10.5). Absorbance was measured at 515 nm using a microplate reader [59].

Concentrations exhibiting over 80% cell viability were selected for further experiments. All assays were performed in triplicate. Cell viability and proliferation were calculated according to Equation (1):

(1) $$\mathrm{Cell}\;\mathrm{viability}\;\left(\%\right)=\frac{{\mathrm{Abs}}_{\;sample}\;-{\;\mathrm{Abs}}_{\;blank}}{{\mathrm{Abs}}_{\;control}\;-{\;\mathrm{Abs}\;}_{blank}}\;\times \;100$$

where ${\mathrm{Abs}}_{\;sample}$ is the absorbance of coffee pulp extract or standard compounds-treated cells, ${\;\mathrm{Abs}}_{\;control}$ is the absorbance of vehicle-treated cells (culture medium), and ${\;\mathrm{Abs}}_{\;blank}$ is the absorbance of wells containing medium only.

### 4.7. Effect of HFDPC-Conditioned Medium on Fibroblast Proliferation

To determine the effect of HFDPC-conditioned medium following extract treatment on human fibroblast proliferation, a conditioned medium approach was used. In brief, HFDPCs were seeded at a density of 1 × 10^5^ cells/mL in 96-well microplates and allowed to adhere for 24 h. After attachment, cells were treated with the coffee pulp extract and standard compound (minoxidil) at concentrations of 0.125 mg/mL for an additional 24 h. Vehicle-treated cells (culture medium) served as the control. Following treatment, culture supernatants were collected and centrifuged at 300× *g* for 3 min at 4 °C to remove cellular debris. Afterward, the cell-free supernatants were then filtered through 0.22 µm syringe filters to obtain sterile conditioned medium and used immediately or stored at 4 °C for no longer than 24 h.

In parallel, human fibroblast cells were seeded at a density of 1 × 10^5^ cells/mL in 96-well microplates and allowed to adhere for 24 h. After attachment, the culture medium was replaced with the collected conditioned medium from treated HFDPCs. Following 24 h incubation with conditioned medium, human fibroblast cell proliferation was assessed using the SRB assay [4]. Cell proliferation was calculated according to Equation (2).

### 4.8. TBT-Induced Reduction in HFDPC Viability Assay

The effects of coffee pulp extracts on HFDPC viability following TBT exposure were assessed using an SRB-based viability assay adapted from [60]. HFDPCs were plated at a density of 1 × 10^5^ cells/mL in 96-well plates and allowed to adhere for 24 h prior to treatment. To induce potassium channel blockade, cells were exposed to 2.5 mM tolbutamide (TBT) for 2 h, after which the medium was replaced with fresh medium containing either coffee pulp extracts or minoxidil as a positive control (each at 0.125 mg/mL), and incubated for a further 24 h. Cell viability was subsequently determined using the SRB assay, and the percentage of viable cells was calculated according to Equation (2).

### 4.9. Anti-Inflammatory Activity Assay

Inflammatory responses in RAW 264.7 and HFDPCs were assessed by quantifying nitric oxide (NO) production via nitrite accumulation in the culture supernatant, using a Griess reaction-based colorimetric assay [4]. Both cell types were seeded at 1 × 10^5^ cells/mL and allowed to adhere for 24 h. Cells were pre-treated with either the coffee pulp extracts, diclofenac sodium (positive control) (each at 0.125 mg/mL), or serum-free medium (untreated control) for 2 h, after which inflammatory responses were triggered by exposure to LPS (1 µg/mL) for a further 24 h. Collected supernatants were reacted with Griess reagent, and nitrite concentrations were interpolated from a sodium nitrite calibration curve (0.01–50 µM).

### 4.10. Reactive Oxygen Species Production Inhibition (H_2_DCFDA Assay)

To determine the intracellular ROS scavenging ability of test compounds, ROS levels were quantified using the fluorescent probe H_2_DCFDA [61]. HFDPC cells were seeded in a dark, clear-bottom 96-well plate at 1 × 10^5^ cells/mL and cultured for 24 h prior to treatment. Cells were then pre-incubated with either the coffee pulp extracts, L-ascorbic acid (used as a positive control) (each at 0.125 mg/mL), or serum-free medium (blank control) for 30 min prior to exposure to hydrogen peroxide (H_2_O_2_) at a final concentration of 10^−5^ M, followed by a further 24 h incubation. After incubation, the culture medium was removed, and the cells were washed with PBS. The cells were then incubated with H_2_DCFDA solution using a Reactive Oxygen Species (ROS) Assay Kit (DCFDA/H_2_DCFDA, Abcam, Cambridge, UK; ab113851) according to the manufacturer’s instructions. Fluorescence intensity was subsequently measured at an excitation/emission wavelength of 485/535 nm to determine intracellular ROS levels.

### 4.11. Thiobarbituric Acid-Reactive Substances (TBARS) Assay

Intracellular oxidative stress was evaluated in HFDPCs through quantification of malondialdehyde (MDA), a key end-product of lipid peroxidation, using the TBARS assay [42]. Cells were plated in 6-well plates at 1 × 10^5^ cells/mL and cultured for 24 h before treatment with either the coffee pulp extract or L-ascorbic acid (positive control) (each at 0.125 mg/mL) or serum-free medium (untreated control) for 24 h. Oxidative stress was subsequently induced by treatment with 100 µM H_2_O_2_ for 2 h. Cells were then lysed in a reaction mixture containing 1% Triton X-100, 0.6% thiobarbituric acid (TBA), and 15% trichloroacetic acid (TCA), heated at 100 °C for 10 min, and immediately quenched at −80 °C for 10 min. MDA levels were determined spectrophotometrically at 532 nm and expressed relative to untreated controls.

### 4.12. Gene Expression Analysis

To evaluate the molecular effects of the coffee pulp extract on hair regeneration-related pathways, semi-quantitative RT-PCR was employed to assess gene expression across three functional categories: (i) androgen metabolism (*SRD5A1* and *SRD5A2*), (ii) transforming growth factor-beta signaling (*TGF-β1*), and (iii) hair follicle growth and regeneration pathways, including Wnt/β-catenin signaling (*CTNNB1*), Sonic Hedgehog signaling (*SHH*, *SMO*, and *GLI1*), and angiogenesis (*VEGF*). Category (i) was assessed in both DU-145 and HFDPC cells, whereas categories (ii) and (iii) were evaluated in HFDPC cells only.

In brief, cells were seeded at 1 × 10^5^ cells/mL and cultured for 24 h, followed by treatment with the coffee pulp extract or respective standard controls—dutasteride, finasteride, minoxidil, and purmorphamine—at 0.125 mg/mL for an additional 24 h. RNA extraction, cDNA synthesis, PCR amplification, and gel imaging were performed as previously described [42], with primer sequences for all target genes and the housekeeping gene *GAPDH* provided in Supplementary Table S2. Gene expression levels were normalized to *GAPDH* and reported as fold changes relative to vehicle-treated controls.

### 4.13. Statistical Analysis

The data are presented as mean ± standard deviation (SD). One-way analysis of variance (ANOVA) was applied to assess differences among groups, followed by Tukey’s post hoc test for multiple comparisons. Statistical significance was set at *p* < 0.05. All analyses were performed using IBM SPSS Statistics (version 23.0; IBM Corp., Armonk, NY, USA)**.** For fermentation kinetics data (TPC and antioxidant activities across the 120 h fermentation period), two-way ANOVA (treatment × time) was applied, followed by Tukey’s HSD post hoc test; capital letters denote significant differences between treatment groups at the same time point, and lowercase letters denote significant differences within the same treatment group across time (*p* < 0.05). For all cell-based and gene expression assays, one-way ANOVA followed by Tukey’s HSD post hoc test was used to compare treatment groups at a single time point. All analyses were performed using IBM SPSS Statistics (version 23.0; IBM Corp., Armonk, NY, USA).

## 5. Conclusions

Mixed-culture fermentation with *S. cerevisiae* and *L. plantarum* generated a chemically and functionally distinct coffee pulp extract. Untargeted metabolomic analysis revealed treatment-dependent remodeling, with LP-CP exhibiting the largest number of significantly altered metabolite features, while MIX-CP showed a predominantly upward pattern among its significantly altered features. MIX-CP also contained comparatively high levels of selected polyphenols, particularly rosmarinic acid and quercetin. In cell-based models, MIX-CP promoted HFDPC proliferation at a sub-cytotoxic concentration, increased fibroblast proliferation in conditioned medium, partially protected HFDPC viability under pharmacological potassium-channel inhibition, and reduced H_2_O_2_-induced intracellular ROS accumulation and lipid peroxidation. At the transcriptional level, MIX-CP decreased *SRD5A1*, *SRD5A2*, and *TGFB1* expression while increasing the expression of selected genes associated with Wnt/β-catenin, Sonic Hedgehog, and angiogenic signaling. Collectively, these findings indicate that the biological effects of fermented coffee pulp were not explained by TPC alone and may reflect qualitative differences in the metabolite profiles generated by mixed-culture fermentation. The results also support the potential valorization of coffee pulp as a source of fermentation-derived plant bioactives. Nevertheless, these findings are limited to in vitro cellular and transcript-level observations, and certain assays—such as the conditioned-medium fibroblast proliferation model—could not fully separate HFDPC-derived paracrine effects from residual direct effects of the extract. Further studies are required to identify the principal active metabolites, incorporate direct-exposure controls, validate pathway activity at the protein and functional levels, and assess efficacy in three-dimensional hair follicles, in vivo, and in clinical models before any cosmeceutical or therapeutic claims can be made.

## Figures and Tables

**Table 1:** Number and proportion of metabolite features showing increased or decreased abundance in fermented coffee pulp extracts compared with the unfermented control without statistical filtering.

Group	Up-Abundance (%)	Down-Abundance (%)	Total Features
LP-CP vs. CP	1639 (55.54%)	1312 (44.46%)	2951
SC-CP vs. CP	1857 (62.92%)	1094 (37.08%)	2951
MIX-CP vs. CP	2397 (81.23%)	554 (18.77%)	2951

Abbreviations: CP, unfermented coffee pulp; LP-CP, *Lactobacillus plantarum*-fermented coffee pulp; SC-CP, *Saccharomyces cerevisiae*-fermented coffee pulp; MIX-CP, mixed-culture fermented coffee pulp. Up- and down-abundance were defined based on fold-change direction (ratio > 1 or < 1, respectively), considering all 2951 retained metabolite features.

**Table 2:** Number and proportion of significantly altered metabolite features in fermented coffee pulp extracts compared with the unfermented control under stringent statistical criteria. Significant features were defined as those with adjusted *p*-value < 0.01 and fold change >2 (upregulated) or <0.5 (downregulated). Percentages represent the proportion of up- or downregulated features within the total significantly altered features for each comparison.

Group	Up-Abundance (%)	Down-Abundance (%)	Total Significantly Altered, *n*
LP-CP vs. CP	666 (65.29%)	354 (34.71%)	1020
SC-CP vs. CP	94 (59.49%)	64 (40.51%)	158
MIX-CP vs. CP	238 (84.10%)	45 (15.90%)	283

Abbreviations: CP, unfermented coffee pulp; LP-CP, *Lactobacillus plantarum*-fermented coffee pulp; SC-CP, *Saccharomyces cerevisiae*-fermented coffee pulp; MIX-CP, mixed-culture fermented coffee pulp.

**Table 3:** Polyphenol composition (mg/g extract) of unfermented and fermented coffee pulp extracts determined by HPLC analysis.

Polyphenol	Extracts (mg/g Extract)			
	CP	LP-CP	SC-CP	MIX-CP
Gallic acid	6.68 ± 0.14	7.74 ± 0.07	6.99 ± 0.00	7.51 ± 0.02
Chlorogenic acid	31.23 ± 0.52	28.65 ± 2.81	11.60 ± 0.86	9.00 ± 1.21
Catechin	34.54 ± 1.30	55.58 ± 2.19	25.57 ± 0.65	31.39 ± 1.03
Epicatechin	40.12 ± 1.76	76.02 ± 0.36	27.15 ± 0.48	36.34 ± 1.56
Caffeic acid	129.93 ± 3.13	78.60 ± 1.19	66.29 ± 6.02	96.93 ± 0.01
*p* -coumaric acid	ND	0.28 ± 0.01	ND	0.25 ± 0.03
Rosmarinic acid	0.71 ± 0.18	0.88 ± 0.30	ND	3.22 ± 0.05
*o* -coumaric acid	0.62 ± 0.01	4.14 ± 0.18	4.01 ± 0.13	3.92 ± 0.02
Quercetin	13.96 ± 0.04	18.76 ± 1.50	10.63 ± 0.03	21.75 ± 0.23

Abbreviations: CP, unfermented coffee pulp; LP-CP, *Lactobacillus plantarum*-fermented coffee pulp; SC-CP, *Saccharomyces cerevisiae*-fermented coffee pulp; MIX-CP, mixed-culture fermented coffee pulp; ND, not detected.

## Data Availability

The original contributions presented in this study are included in the article/Supplementary Materials. Further inquiries can be directed to the corresponding author.

## Supplementary Materials

The following supporting information can be downloaded at https://www.mdpi.com/article/10.3390/ijms27177865/s1.
