# Supplementary material for: Mixed-Culture Fermentation of Coffee Pulp Induces Metabolomic Changes and Enhances Multi-Target Bioactivity Relevant to Androgenetic Alopecia
Source: Int J Mol Sci. 2026 Sep 2;27(17):7865. doi: 10.3390/ijms27177865 (PMC13565882; doi:10.3390/ijms27177865)
Supplement: Supplementary file 1 [file ijms-27-07865-s001.zip › ijms-4487217-supplementary/Supplementary Table S1- Cell viability.pdf]

Table S1: Cell viability (%) of HFDPC, RAW 264.7, hTERT, and DU-145 cells treated with fermented and unfermented coffee pulp extracts via SRB assay

HFDPC		Concentration (mg/mL)									
		**2.000**	**1.000**	**0.500**	**0.250**	**0.125**	**0.063**	**0.031**	**0.016**	**0.008**	**0.004**
**CP**	**Viability**	49.74	52.82	57.52	64.48	80.97	82.31	99.24	101.07	102.56	99.81
	**S.D.**	1.55	1.03	1.88	1.37	1.92	1.14	1.66	2.65	1.79	1.48
**LC-CP**	**Viability**	47.85	51.62	59.73	71.28	83.15	90.42	100.67	104.12	99.85	100.62
	**S.D.**	1.09	1.74	1.32	1.86	1.15	1.63	1.98	1.83	1.51	1.07
**SC-CP**	**Viability**	49.21	53.04	61.15	73.62	84.9	91.78	100.23	102.52	100.05	100.78
	**S.D.**	1.83	1.19	2.04	1.46	1.68	1.01	1.57	3.22	1.93	1.36
**MIX-CP**	**Viability**	50.37	54.18	62.44	78.09	85.63	92.35	96.89	146.38	100.21	101.14
	**S.D.**	1.16	1.62	1.08	1.97	1.29	1.75	1.04	2.98	1.38	1.81

RAW 264.7		Concentration (mg/mL)									
		**2.000**	**1.000**	**0.500**	**0.250**	**0.125**	**0.063**	**0.031**	**0.016**	**0.008**	**0.004**
**CP**	**Viability**	54.87	59.63	67.92	82.08	88.45	93.27	96.58	98.63	99.87	100.42
	**S.D.**	1.63	1.11	1.84	1.29	1.97	1.06	1.71	1.48	1.15	1.89
**LC-CP**	**Viability**	57.35	62.08	80.15	93.52	99.85	94.61	97.35	99.08	100.23	100.71
	**S.D.**	1.34	1.78	1.06	1.52	2.03	1.24	1.66	1.09	1.91	1.41
**SC-CP**	**Viability**	59.72	63.45	81.94	85.31	91.28	95.42	97.87	99.31	100.42	100.94
	**S.D.**	1.87	1.32	1.59	1.01	1.74	1.13	1.98	1.26	1.65	1.08
**MIX-CP**	**Viability**	55.29	60.14	78.53	82.76	89.12	93.94	96.75	98.79	100.08	100.55
	**S.D.**	1.05	1.69	1.16	1.88	1.31	1.72	1.03	1.57	1.94	1.22

hTERT		Concentration (mg/mL)									
		**2.000**	**1.000**	**0.500**	**0.250**	**0.125**	**0.063**	**0.031**	**0.016**	**0.008**	**0.004**
**CP**	**Viability**	52.17	54.6	61.89	83.23	87.93	99.57	100.45	100.62	101.91	100.78
	**S.D.**	1.34	1.72	2.05	1.18	1.63	1.87	1.29	1.51	1.06	1.94
**LC-CP**	**Viability**	58.32	62.15	70.44	83.67	89.21	94.05	97.18	98.73	100.12	100.85
	**S.D.**	1.47	1.09	1.83	1.36	1.58	1.02	1.71	1.24	1.95	1.13
**SC-CP**	**Viability**	56.9	61.03	69.28	84.52	90.15	93.87	96.94	99.02	100.33	101.05
	**S.D.**	1.61	1.28	1.99	1.45	1.07	1.82	1.16	1.69	1.33	1.91
**MIX-CP**	**Viability**	59.14	63.28	81.65	85.03	91.42	95.11	97.56	99.24	100.48	100.92
	**S.D.**	1.22	1.78	1.05	1.53	1.96	1.31	1.64	1.11	1.87	1.42

DU-145		Concentration (mg/mL)									
		**2.000**	**1.000**	**0.500**	**0.250**	**0.125**	**0.063**	**0.031**	**0.016**	**0.008**	**0.004**
**CP**	**Viability**	57.23	61.85	69.92	83.14	89.76	94.32	97.05	98.94	100.27	100.63
	**S.D.**	1.42	1.85	2.06	1.23	1.01	1.67	1.34	1.52	1.91	1.08
**LC-CP**	**Viability**	55.68	60.32	68.15	82.47	88.93	93.68	96.82	98.56	99.94	100.38
	**S.D.**	1.19	1.56	1.98	1.11	1.73	1.05	1.62	1.28	1.86	1.44
**SC-CP**	**Viability**	58.94	62.71	80.83	89.29	7.54	94.87	97.43	99.12	100.36	100.79
	**S.D.**	1.77	1.03	1.49	1.92	1.14	1.68	1.31	1.06	1.83	1.57
**MIX-CP**	**Viability**	56.42	60.89	79.37	83.85	89.62	94.15	97.28	98.87	100.15	100.52
	**S.D.**	1.09	1.64	2.02	1.37	1.79	1.02	1.55	1.21	1.96	1.33
