# Supplementary material for: Mixed-Culture Fermentation of Coffee Pulp Induces Metabolomic Changes and Enhances Multi-Target Bioactivity Relevant to Androgenetic Alopecia
Source: Int J Mol Sci. 2026 Sep 2;27(17):7865. doi: 10.3390/ijms27177865 (PMC13565882; doi:10.3390/ijms27177865)
Supplement: Supplementary file 1 [file ijms-27-07865-s001.zip › ijms-4487217-supplementary/Supplementary Table S2- Primer sequences .pdf]

Table S2: Primer sequences of androgen metabolism genes (*SRD5A1* and *SRD5A2*), transforming growth factor-beta (*TGFB1*), Wnt/β-catenin (*CTNNB1*), Sonic Hedgehog (*SHH*, *SMO*, *GLI1*), and angiogenesis (*VEGF*), alongside the *GAPDH* housekeeping gene.

Target Category	Pathway	Gene	Accession Number	Forward Sequence	Reverse Sequence
Hair loss induction	5α-reductase	*SRD5A1*	NM_001047.4	AGCCATTGTGCAGTGTATGC	AGCCTCCCCTTGGTATTTTG
		SRD5A2	NM_000348.4	TGAATACCCTGATGGGTGG	CAAGCCACCTTGTGGAATC
		SRD5A3	NM_024592.5	TCCTTCTTTGCCCAAACATC	TCCTTCTTTGCCCAAACATC
	Transforming growth factor beta 1	*TGFB1*	NM_00600.7	AACCCACAACGAAATCTATG	CTTTTAACTTGAGCCTCAGC
Hair growth promotion	Sonic Hedgehog	*SHH*	NM_000193.4	AAAAGCTGACCCCTTTAGCC	GCTCCGGTGTTTTCTTCATC
		SMO	NM_005631.5	GAAGTGCCCTTGGTTCGGACA	CCGCCAGTCAGCCACGAAT
		GLI1	NM_005269.3	GCAGGGAGTGCAGCCAATACAG	GAGCGGCGGCTGACAGTATA
	Wnt/β-catenin	*CTNNB1*	NM_001330729.2	CCCACTAATGTCCAGCGTTT	AACCAAGCATTTTCACCAGG
	Angiogenesis	*VEGF*	NM_001025366.3	CTACCTCCACCATGCCAAGT	GCGAGTCTGTGTTTTTGCAG
Internal control		*GAPDH*	NM_001289745.3	GGAAGGTGAAGGTCGGAGTC	CTCAGCCTTGACGGTGCCATG
